# Kernel Bayesian logistic tensor decomposition with automatic rank determination for predicting multiple types of miRNA-disease associations

**DOI:** 10.1371/journal.pcbi.1012287

**Published:** 2024-07-08

**Authors:** Yingjun Ma, Yuanyuan Ma

**Affiliations:** 1 School of Mathematics and Statistics, Xiamen University of Technology, Xiamen, China; 2 School of Computer Engineering, Hubei University of Arts and Science, Xiangyang, China; Robert Koch Institute: Robert Koch Institut, GERMANY

## Abstract

Identifying the association and corresponding types of miRNAs and diseases is crucial for studying the molecular mechanisms of disease-related miRNAs. Compared to traditional biological experiments, computational models can not only save time and reduce costs, but also discover potential associations on a large scale. Although some computational models based on tensor decomposition have been proposed, these models usually require manual specification of numerous hyperparameters, leading to a decrease in computational efficiency and generalization ability. Additionally, these linear models struggle to analyze complex, higher-order nonlinear relationships. Based on this, we propose a novel framework, KBLTDARD, to identify potential multiple types of miRNA–disease associations. Firstly, KBLTDARD extracts information from biological networks and high-order association network, and then fuses them to obtain more precise similarities of miRNAs (diseases). Secondly, we combine logistic tensor decomposition and Bayesian methods to achieve automatic hyperparameter search by introducing sparse-induced priors of multiple latent variables, and incorporate auxiliary information to improve prediction capabilities. Finally, an efficient deterministic Bayesian inference algorithm is developed to ensure computational efficiency. Experimental results on two benchmark datasets show that KBLTDARD has better Top-1 precision, Top-1 recall, and Top-1 F1 for new type predictions, and higher AUPR, AUC, and F1 values for new triplet predictions, compared to other state-of-the-art methods. Furthermore, case studies demonstrate the efficiency of KBLTDARD in predicting multiple types of miRNA-disease associations.

## Author summary

Many studies have shown that miRNAs contribute to the onset and development of disease through a variety of potential mechanisms. Therefore, predicting multi-category associations between miRNAs and diseases is helpful for the diagnosis and treatment of complex diseases. Different from existing methods, our KBLTDARD combines the logistic tensor decomposition and Bayesian methods, realises the automatic search of hyperparameters by introducing sparse induction priors of multiple latent variables, and incorporates auxiliary information to improve the predictive ability of the Bayesian model. Unlike the simple overfitting of point estimation and the slow convergence of MCMC, we developed an efficient deterministic Bayesian inference algorithm to ensure solution efficiency. We performed 5-fold cross-validation to evaluate the performance of KBLTDARD, which can outperform other state-of-the-art methods. Moreover, the average AUC value of KBLTDARD for the prediction of 447 diseases reached 0.8165. For the prediction of miRNAs and association types of four common diseases, 17, 16, 16 and 17 of the top 20 triples were verified from the literature.

## Introduction

MicroRNAs (miRNAs) are a group of small noncoding RNAs that play important roles in many biological processes [1]. They have the ability to inhibit or promote gene expression, thereby affecting protein synthesis. As a result, dysregulation of miRNAs is associated with various biological processes and diseases [2–4]. The identification of disease-related miRNAs is highly significant for studying disease pathogenesis and drug development. In the past, biological experiments were utilized for this purpose, but such methods were not only time-consuming and laborious, but also inadequate for large-scale detection of the miRNA-disease association [5]. With the development of high-throughput sequencing technology, many related databases for miRNA and disease research have been established. Notably, DIANA-TarBase [6], miRTarBase [7], and miRWalk [8] offer a vast collection of miRNA-gene associations. MiRbase [9] and MiREDiBase [10], on the other hand, furnish sequence data and miRNA editing sites, respectively. The Comparative Toxicogenomics Database (CTD) [11] gathers a vast array of biological entities and associated information, including diseases, genes, phenotypes, and chemical compounds. These databases have significantly broadened our comprehension of miRNA functions and their regulatory mechanisms, serving as a foundation for constructing computational models to anticipate potential miRNA-disease associations.

In recent years, numerous models for predicting miRNA-disease associations have been proposed. Tang et al. [12] developed a multi-channel graph convolutional network, which utilized a GCN encoder to capture features under different views, and augmented the learned prediction representation by multi-channel attention. Ma et al. [13] proposed a graph autoencoder model to address the over-smoothing problem of the GNN method, which employed a graph encoder to splice aggregate feature embeddings and self-feature embeddings, and adopted a bilinear decoder for link prediction. In previous research, to obtain higher quality similarity networks, we introduced kernel neighborhood similarity into multi-network bidirectional propagation, which effectively integrates multi-network information to improve prediction performance [14]. Li et al. [5] integrated GCN, CNN and the Squeeze Excitation Network (GCSENet) to devise a novel prediction model for miRNA-disease associations. The model utilizes GCN to gather the features from the miRNA-disease-gene heterogeneous network, performs convolutional operations via CNN, and determines the importance of each feature channel by employing SENet’s squeeze and excite blocks.

Most of the aforementioned techniques concentrate on foreseeing a binary association between miRNA and disease. Nevertheless, mounting evidence indicates that the malfunction of miRNAs triggers disease through various conceivable mechanisms [15,16]. On the one hand, only certain types of miRNAs are the cause of disease. For instance, targeted deletion of heart and muscle-specific miR-1-2 results in defects in cardiac morphogenesis, such as ventricular septal defects, and high mortality before and after birth [17]. On the other hand, the same miRNA can also cause the same disease through a different set of pathways. For example, miR-146a can directly target SMAD4, thereby regulating cell proliferation and apoptosis and playing a role in the onset and development of gastric cancer [18]. The ectopic expression of miR-146a inhibits the migration and invasion of gastric cancer cells, and down-regulates the expression of EGFR and IRAK1 [19]. Therefore, identifying miRNA-disease associations and associated types will enhance our understanding of the pathogenesis of diseases related to miRNA dysregulation on a deeper level.

In recent years, researchers have placed greater emphasis on identifying different types of miRNA-disease associations. Chen et al. [20] first proposed a restricted Boltzmann machine model (RBMMMDA) for predicting associations of miRNAs, diseases, and related types. However, RBMMMDA neglects the use of auxiliary information, which limits its predictive power to some extent. Inspired by the successful application of the existing tensor decomposition method in studying high-order biological relationships [21], Huang et al. [22] initially introduced the CANDECOMP/PARAFAC (CP) decomposition technique to multi-type miRNA-disease association prediction, coupled with biological similarity serving as a constraint to enhance prediction accuracy. Subsequently, Wang et al. [23] developed a NMCMDA method utilizing end-to-end data-driven learning for the prediction of multi-category miRNA-disease associations. To address the challenges of the current tensor decomposition model, which easily falls into local minima and produces false-negative samples, Dong et al. [24] developed a novel multi-type miRNA-disease association prediction model by integrating hypergraph learning and tensor weighting into non-negative tensor decomposition. Due to the intrinsic lack of completeness and noise in miRNA-disease-type datasets, Yu et al. [25] combined tensor decomposition and label propagation, employed robust principal component analysis on tensors to obtain low-rank prediction tensors, and utilized label propagation to transfer information. While the previously developed methods have yielded success in multi-type disease-related miRNAs prediction tasks, there are still limitations. Firstly, calculating miRNA similarities with the help of known miRNA-disease associations will cause the model to rely on the known associations. Secondly, both CP decomposition and non-negative tensor decomposition are linear models, which hinders the ability to identify complex nonlinear relationships among miRNAs, diseases and association types. Finally, the above models include numerous hyperparameters requiring adjustment, affecting both model computational efficiency and generalization ability.

To address the above challenges, we propose a novel computational model called Kernel Bayesian Logistic Tensor Decomposition with Automatic Rank Determination (KBLTDARD) for predicting different types of miRNA-disease associations. Firstly, to reduce dependence on known associations and enhance network precision, we construct the functional similarity of miRNAs from other data sources beyond miRNA-disease-type, and fused multiple similarities to obtain more accurate miRNA(disease) similarity. Secondly, to ensure nonlinear learning ability and avoid tedious hyperparameter debugging, we build hierarchical probability model to formulate logical tensor decomposition, and employ full Bayesian treatment to sparse induction priors of multiple latent variables, enabling automatic search of hyperparameters. Finally, a highly efficient deterministic Bayesian inference algorithm was developed to ensure solution efficiency. Experimental results indicate that LTDSSL outperforms other state-of-the-art methods in predicting multiple types of miRNA-disease associations with high accuracy.

## Materials and methods

### Method review

**Fig 1:**
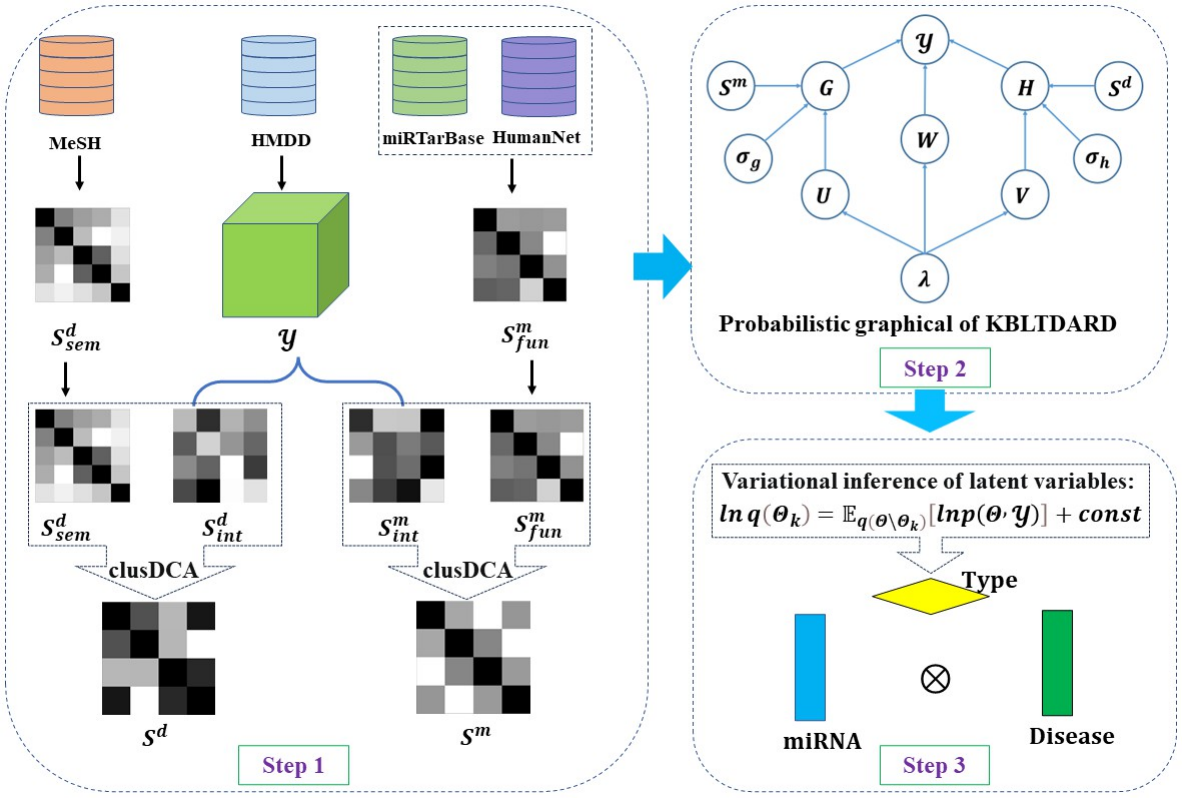
The workflow of our proposed KBLTDARD model.

In this section, we propose a new computational model called KBLTDARD for predicting miRNA-disease-type associations, which mainly consists of three steps (shown in Fig 1). Firstly, multiple similarities of miRNA (disease) are calculated and fused to obtain a more accurate miRNA (disease) similarity network (shown in step 1 of Fig 1). Secondly, a Bayesian framework of logistic tensor decomposition is established, and the auxiliary information of miRNA (or disease) and the prior probability of latent variables are introduced to construct the probabilistic graphical model of KBLTDARD (shown in step 2 of Fig 1). Finally, the Bayesian variational inference framework for KBLTDARD is established to realize the prediction of potential miRNA-disease-type associations (shown in step 3 of Fig 1).

### Dataset

The Human MiRNA Disease Database (HMDD) contains extensive data on experimentally validated human miRNA-disease associations [15,26]. Many computational models utilize HMDD to establish benchmark data sets for execution studies [22–25,27]. To facilitate comparison, we utilize two widely used multi-type miRNA-disease data sets (HMDD v2.0 and HMDD v3.2) established by Huang et al. [22] as benchmark data sets. Specifically, HMDD v2.0 classifies miRNA-disease associations into four types based on evidence from circulation, epigenetics, genetics, and target, containing 1,675 associations for 324 miRNAs and 169 diseases under the four types, with a density of 0.681% in this dataset. HMDD v3.2 contains 16,341 associations of 713 miRNAs and 447 diseases under five types: circulation, epigenetics, genetics, target, and tissue, with a density of 1.025% in the dataset. To obtain additional auxiliary information beyond the associated data, Huang et al. also downloaded disease descriptors from the Medical Subject Headings (MeSH) and calculated the semantic similarity of the diseases [28].

Furthermore, to avoid dependence on known miRNA-disease-type associations, according to previous studies [12, 14], we extracted miRNA-gene associations from miRTarBase Release 8.0 [7] and functional association probabilities of genes from HumanNet [29]. Then, the functional similarity of miRNAs can be obtained by combining the above two kinds of association information.

### Tensor construction

Given a set of miRNAs $M=\left\{m_{1},m_{2},\cdots ,m_{I}\right\}$, a set of diseases $D=\left\{d_{1},d_{2},\cdots ,d_{J}\right\}$, and a set of association types $T=\left\{t_{1},t_{2},\cdots ,t_{K}\right\}$. Then, all associations of miRNAs, diseases and types can be described by the third-order tensor $Y\in {\left\{0,1\right\}}^{I\times J\times K}$. The (*i*, *j*, *k*) element in tensor Y is recorded as $Y_{i,j,k}$, which represents the relationship between miRNA *m*_*i*_ and disease *d*_*j*_ under association type *t*_*k*_. When *m*_*i*_ and *d*_*j*_ are associated under *t*_*k*_, $Y_{i,j,k}=1$; otherwise, $Y_{i,j,k}=0$. The matrix *Y*_∷*k*_ is the *k*th frontal slice of Y, representing the *k*-th type of miRNA-disease association. Previous studies on miRNA-disease associations [5,12,30,31] ignored the impact of association types and were equivalent to research on a certain association type.

Although some associations have been discovered and validated, there still exists many associations that have not been verified, and inferring these potential associations may improve our understanding of the pathogenic mechanisms of different types of miRNAs. To this end, our goal is the prediction of potential miRNA-disease-type triples, which is the tensor completion problem. However, since there are few known associations, the tensor Y is very sparse and contains very limited useful information. Therefore, extracting the auxiliary information of the miRNA and the disease is an effective way to improve the performance of prediction.

### MiRNA functional similarity

To prevent dependency on known miRNA-disease associations, we exploited known miRNA-gene associations and gene similarity to calculate miRNA functional similarity. Referring to previous studies [12, 32], let *LLS*(*g*_*i*_, *g*_*j*_) denote the association log-likelihood score between gene *g*_*i*_ and *g*_*j*_ obtained from HumanNet. Then, the similarity *S*(*g*_*i*_, *g*_*j*_) between *g*_*i*_ and *g*_*j*_ is

$$S\left(g_{i},g_{j}\right)=\left\{\begin{array}{c} \;0\;e\left(g_{i},g_{j}\right)\notin \text{HumanNet}\\ 1\;g_{i}=g_{j}\\ \frac{LLS\left(g_{i},g_{j}\right)-LLS_{min}}{LLS_{max}-LLS_{min}}\;e\left(g_{i},g_{j}\right)\notin \text{HumanNet} \end{array}\right.$$ (1)

where *e*(*g*_*i*_, *g*_*j*_) is the edge between genes *g*_*i*_ and *g*_*j*_. *LLS*_*min*_ and *LLS*_*max*_ denote the minimum and maximum log-likelihood score in HumanNet, respectively. Let *G*_*i*_ and *G*_*j*_ denote the gene sets associated with miRNA *m*_*i*_ and *m*_*j*_ respectively, then the functional similarity can be calculated as follows:

$$S\left(m_{i},m_{j}\right)=\frac{\sum_{g\in G_{i}}S\left(g_{i},G_{j}\right)+\sum_{g\in G_{j}}S\left(g_{i},G_{i}\right)}{\left|G_{i}\right|+\left|G_{j}\right|}$$ (2)

where *S*(*g*,*G*) = *max*{*S*(*g*, *g*_*i*_) |*g*_*i*_ ∈ *G*}, |*G*_*i*_| and |*G*_*j*_| represent the number of genes contained in *G*_*i*_ and *G*_*j*_, respectively.

In summary, for each benchmark dataset, we obtain miRNA-disease-type tensor Y, disease semantic similarity $S_{sem}^{d}$ and miRNA functional similarity $S_{fun}^{m}$.

### Network integration

The known tensor Y also contains important information, which can be an important supplement to the similarity of miRNA (disease) [22,27]. Dong et al. [27] employed Y to construct a miRNA-disease association matrix, and then calculated the Gaussian similarity of miRNAs, which ignored the influence of association type. Therefore, to retain the information of miRNA, disease and association types simultaneously, we extract features directly from Y and fuse them to obtain a more accurate similarity network.

Let matrix $Y^{\left(1\right)}\in R^{I\times JK}$ represent the mode-1 matrixization of Y, that is, project the disease and type dimensions of Y onto the columns of *Y*^(1)^. Then, $Y_{i.}^{\left(1\right)}$ is the *i*th row of *Y*^(1)^, which represents the interaction profile feature of the *i*-th miRNA. Similarly, let matrix $Y^{\left(2\right)}\in R^{J\times IK}$ represent the mode-2 matrixization of Y, which also represents the interaction profile feature of diseases. Since *Y*^(1)^ and *Y*^(2)^ are both high-dimensional and sparse association matrices, which inevitably contain noise. Therefore, we first employ non-negative double singular value decomposition (NNDSVD) [33] for dimensionality reduction to obtain the non-negative low-dimensional features *F*^*m*^ and *F*^*d*^ of miRNA and disease respectively.

Then, we adopt Kernel Soft-neighborhood Similarity (KSNS) to build the similarity network. Referring to previous studies [14,34–39], KSNS hierarchically integrates neighborhood information and mines nonlinear relationships of samples, and has been well applied to the prediction of various types of biological interactions. Therefore, according to *F*^*m*^ and *F*^*d*^, the interaction profile similarities $S_{int}^{m}$ and $S_{int}^{d}$ of miRNAs and diseases are obtained by KSNS, respectively.

Finally, we obtained two types of miRNA similarity ($S_{fun}^{m}$ and $S_{int}^{m}$) and two types of disease similarity ($S_{sem}^{d}$ and $S_{int}^{d}$), which measured the similarity relationship of miRNA (or diseases) from different perspectives. Referring to previous studies [36,40], we utilized clusDCA to fuse $S_{fun}^{m}$ and $S_{int}^{m}$ to obtain the integrated similarity *S*^*m*^ of miRNAs, and fused $S_{sem}^{d}$ and $S_{int}^{d}$ to obtain the integrated similarity *S*^*d*^ of diseases.

### KBLTDARD

In previous research, we established a new tensor decomposition model (LTDSSL), which introduces logistic functions into tensor decomposition to improve nonlinear learning capabilities, showing strong performance in higher-order relation prediction problems [41]. However, LTDSSL requires manually specifying the rank of the tensor and the values of the hyperparameters, without considering the uncertainty information of potential factors. Based on this, this study combines tensor decomposition and Bayesian inference to establish a new tensor decomposition model.

**Fig 2:**
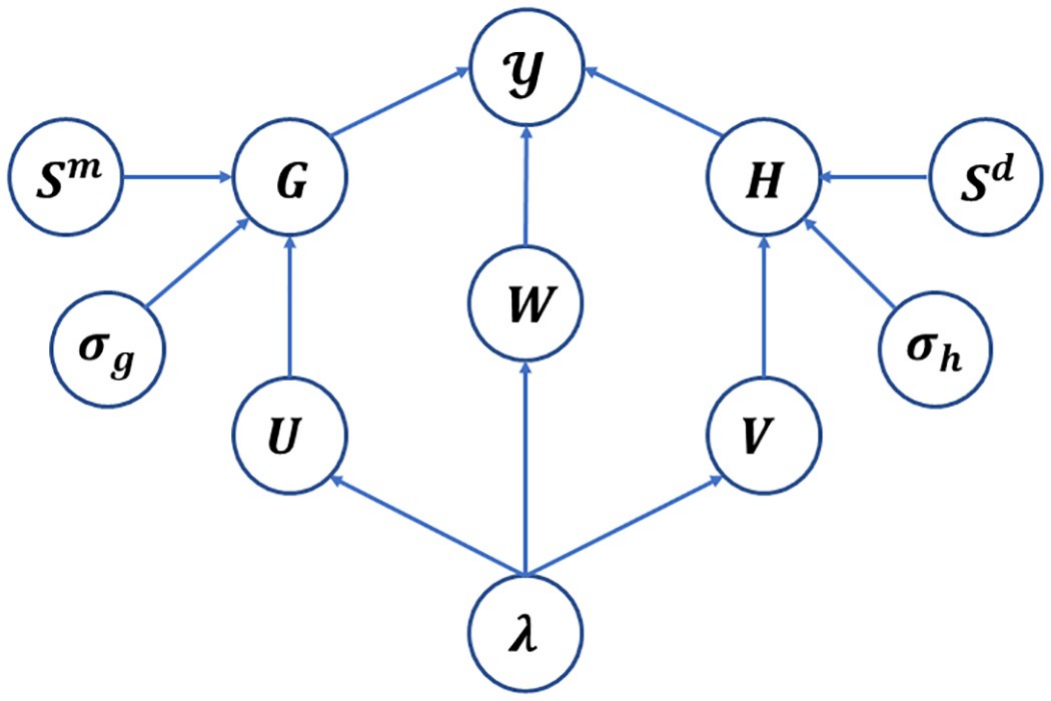
The probability graph framework of KBLTDARD.

Let $G\in R^{I\times R},\;W\in R^{J\times R}$ and $H\in R^{K\times R}$ represent the latent factor matrices of miRNAs, diseases and association types respectively. Then, the association probability $P_{ijk}$ of the *i*th miRNA, *j*th disease, and the *k*th type is as follows:

$$P_{ijk}=\frac{exp\left({\tilde{Y}}_{ijk}\right)}{1+exp\left({\tilde{Y}}_{ijk}\right)}$$ (3)

where $\tilde{Y}=⟦G,W,H⟧\in R^{I\times J\times K}$ is reconstructed tensor, and its (*i*, *j*, *k*)th entry ${\tilde{Y}}_{ijk}$ is $\sum_{r=1}^{R}G_{ir}W_{jr}H_{kr}$. The known miRNA-disease-type triplets are experimentally verified and has higher reliability. Therefore, the weighted logical tensor decomposition model is obtained as follows:

$$P\left(Y|G,W,H\right)=\prod_{i=1}^{I}\prod_{j=1}^{J}\prod_{k=1}^{K}{P_{ijk}}^{cY_{ijk}}{\left(1-P_{ijk}\right)}^{1-Y_{ijk}}$$ (4)

where *c* ≥ 1 represents the importance level parameter. Fig 2 presents the probabilistic graphical model of KBLTDARD with latent variables and corresponding priors.

In Fig 2, the occurrence probability of Y is calculated from *G*, *W* and *H* by (6). The probability distribution of the factor matrix *G* is obtained by $U\in R^{I\times R}$ combined with the miRNA similarity *S*^*m*^, and the probability distribution of the factor matrix *H* is obtained by V combined with the disease similarity *S*^*d*^. *σ*_*g*_, *σ*_*h*_ and ***λ*** are precision parameters. We specify the priors of all latent variables and parameters in this section.

To effectively integrate the auxiliary information, the elements of the factor matrix *G* are independent, and the (*i*, *r*)th element *G*_*i*,*r*_ satisfies the multivariate Gaussian distribution with expectation $S_{i∙}^{\left(m\right)}U_{∙r}$ and precision *σ*_*g*_

$$P\left(G|U,S^{\left(m\right)},\sigma_{g}\right)=\prod_{i=1}^{I}\prod_{r=1}^{R}N\left(G_{i,r}|S_{i\cdot }^{\left(m\right)}U_{\cdot r},{\sigma_{g}}^{-1}\right)$$ (5)

Similarly, let the elements of *H* be independent, and the (*j*, *r*)th element *H*_*j*,*r*_ satisfies the multivariate Gaussian distribution with expectation $S_{j∙}^{\left(d\right)}V_{∙r}$ and precision *σ*_*h*_

$$P\left(H|V,S^{\left(d\right)},\sigma_{h}\right)=\prod_{j=1}^{J}\prod_{r=1}^{R}N\left(H_{j,r}|S_{j\cdot }^{\left(d\right)}V_{\cdot r},{\sigma_{h}}^{-1}\right)$$ (6)

Here, the accuracy parameters *σ*_*g*_ and *σ*_*h*_ of the Gaussian distribution satisfy the Jeffreys prior

$$\begin{array}{c} P\left(\sigma_{g}\right)\propto {\sigma_{g}}^{-1}\\ P\left(\sigma_{h}\right)\propto {\sigma_{h}}^{-1} \end{array}$$ (7)

In general, the effective dimension *R* of the latent space is the tuning parameter, the selection of which is quite challenging and costly. To both infer the value of *R* and avoid overfitting, we introduce automatic rank determination into the priors of *U*, *V* and *W* [42]. Specifically, let each column of *U* be an independent random vector, and whose *r*th column satisfy the multivariate Gaussian distribution with a mean vector **0** and precision matrices *λ*_*r*_ *E*_*I*_

$$P\left(U|\lambda \right)=\prod_{r=1}^{R}N\left(U_{\cdot r}|0,{\lambda_{r}}^{-1}E_{I}\right)$$ (8)

where *E*_*I*_ represents the identity matrix of size *I* × *I*, and *λ*_*r*_ controls the *r*th column of *U*. When *λ*_*r*_ has a large value, *U*_*r*_ approaches 0, indicating that they make little contribution to Y and can be removed from *U*. This process realizes the automatic determination of *R*. Similarly, the *r*th column of *V* satisfies the multivariate Gaussian prior with a mean vector **0** and precision matrices *λ*_*r*_*E*_*J*_

$$P\left(V|\lambda \right)=\prod_{r=1}^{R}N\left(V_{\cdot r}|0,{\lambda_{r}}^{-1}E_{J}\right)$$ (9)

where *E*_*J*_ represents the identity matrix of size *J* × *J*. Similar to *U* and *V*, the prior distribution of *W* is as follows

$$P\left(W|\lambda \right)=\prod_{r=1}^{R}N\left(W_{\cdot r}|0,{\lambda_{r}}^{-1}E_{K}\right)$$ (10)

where *E*_*K*_ represents the identity matrix of size *K* × *K*.

To achieve joint column sparsity of *U*, *V* and *W* [43], we further assign the conjugate Gamma prior to ***λ***

$$P\left(\lambda |\alpha ,\beta \right)=\overset{R}{\underset{r=1}{\prod }}Gamma\left(\lambda_{r}|\alpha ,\beta \right)=\overset{R}{\underset{r=1}{\prod }}\frac{\beta^{\alpha }}{\Gamma \left(\alpha \right)}{\lambda_{r}}^{\alpha -1}e^{-\beta \lambda_{r}}$$ (11)

where, ***λ*** = {*λ*_1_, *λ*_*2*_, ⋯,*λ*_*R*_}, {*α*, *β*} are the two parameters of the Gamma distribution, and we adopt the uninformative prior, that is, *α* = 1, *β* = 1 [38].

For simplicity of notation, all unknown latent variables are collected and denoted together by Θ = {*G*, *H*, *W*, *U*, *V*, ***λ***, *σ*_*g*_, *σ*_*h*_}. The probabilistic graphical model is shown in Fig 2, from which we can easily write the joint distribution of the model as

$$\begin{array}{cc} P\left(Y,\Theta \right)= & P\left(Y|G,H,W\right)P\left(G|U,S^{m},\sigma_{g}\right)P\left(H|V,S^{d},\sigma_{h}\right)\\ & P\left(W|\lambda \right)P\left(U|\lambda \right)P\left(V|\lambda \right)P\left(\lambda \right)P\left(\sigma_{g}\right)P\left(\sigma_{h}\right) \end{array}$$ (12)

Combining the likelihood in (4), the priors of model parameters *G* and *H* in (5) and (6), the prior distributions of *U*, *V* and *W* in (8), (9) and (10), and the hyperpriors in (7) and (11), the logarithmic joint distribution of KBLTDARD can be obtained (see S1 Text of S1 File for details)

$$\begin{array}{c} l\left(\Theta \right)=\overset{I}{\underset{i=1}{\sum }}\overset{J}{\underset{j=1}{\sum }}\overset{K}{\underset{k=1}{\sum }}\left[cY_{ijk}ln\left(\sigma \left({\tilde{Y}}_{ijk}\right)\right)+\left(1-Y_{ijk}\right)Ln\left(1-\sigma \left({\tilde{Y}}_{ijk}\right)\right)\right]\\ -\frac{1}{2}\left[{\left\|\sigma_{g}G-S^{m}U\right\|}_{F}^{2}+\sigma_{h}{\left\|H-S^{d}V\right\|}_{F}^{2}\right]\\ -\frac{1}{2}\left[tr\left(W\Lambda W^{T}\right)+tr\left(U\Lambda U^{T}\right)+tr\left(V\Lambda V^{T}\right)+2\beta tr\left(\Lambda \right)\right]\\ +\left(\frac{IR}{2}-1\right)ln\left(\sigma_{g}\right)+\left(\frac{JR}{2}-1\right)ln\left(\sigma_{h}\right)\\ +\left(\frac{I+J+K}{2}+\alpha -1\right)tr\left(ln\left(\Lambda \right)\right)+const \end{array}$$ (13)

where *Λ* = *diag*(*λ*_1_, *λ*_2_, ⋯, *λ*_*R*_), *ln*(*Λ*) = *diag*(*ln*(*λ*_1_), *ln*(*λ*_2_), ⋯, *ln*(*λ*_*R*_)), and *diag*(**∙**) denotes converting the vector into a diagonal matrix. In (13), *tr*(∙) represents the trace of the square matrix, *const* represents a constant independent of *Θ*, and *ℓ*(*Θ*) represents the logarithmic joint distribution, that is, $lnp\left(\Theta ,Y\right)$. Without losing generality, performing the maximum posterior estimate of Θ by maximizing (13) is somewhat equivalent to optimizing the square error function with regularization applied to the logical tensor decomposition and additional constraints on the regularization parameters.

However, unlike point estimation, our goal is to compute the complete posterior distribution of all variables in *Θ* given the data tensor Y and the similarities (*S*^*m*^ and *S*^*d*^), that is,

$$P\left(\Theta |Y\right)=\frac{P\left(\Theta ,Y\right)}{\int P\left(\Theta ,Y\right)d\Theta }$$ (14)

### Model Inference of KBLTDARD

An exact Bayesian inference in (14) requires integration over all latent variables, which is analytically intractable. Therefore, this study adopts variational inference to calculate the approximate posterior distribution *q*(Θ) of the latent variable [42–44]. The principle of variational inference is to define a parameter distribution group on the latent variable and update the parameters to minimize the Kullback–Leibler (KL) distance between $P\left(\Theta |Y\right)$ and *q*(Θ)

$$\begin{array}{c} {min}_{q\left(\Theta \right)}KL\left(q\left(\Theta \right)|P\left(\Theta |Y\right)\right)=\underset{q\left(\Theta \right)}{min}\left\{\int q\left(\Theta \right)\;ln\frac{q\left(\Theta \right)}{P\left(\Theta |Y\right)}d\Theta \right\}\\ =lnP\left(Y\right)-\underset{q\left(\Theta \right)}{min}\left\{\int q\left(\Theta \right)\;ln\left[\frac{P\left(\Theta ,Y\right)}{q\left(\Theta \right)}\right]d\Theta \right\} \end{array}$$ (15)

where *lnP*(*Y*) is a constant, representing model evidence, and its lower bound is defined as $L\left(q\right)=\int q\left(\Theta \right)\;\text{ln}\;\left\{\frac{P\left(\Theta ,Y\right)}{q\left(\Theta \right)}\right\}\;d\Theta$. With a mean field approximation, *q*(*Θ*) is factorized according to the latent variables as

$$q\left(\Theta \right)=\prod_{k}q\left(\Theta_{k}\right)=q\left(G\right)q\left(H\right)q\left(W\right)q\left(U\right)q\left(V\right)q\left(\lambda \right)q\left(\sigma_{g}\right)q\left(\sigma_{h}\right)$$ (16)

It is worth noting that (16) is the only assumption for the posterior distribution of the latent variable. When other variables are fixed, the approximate logarithmic posterior distribution of the latent variable *Θ*_*k*_ can be accurately obtained

$$ln\;q\left(\Theta_{k}\right)=E_{q\left(\Theta \backslash \Theta_{k}\right)}\left[lnp\left(\Theta ,Y\right)\right]+const$$ (17)

where $E\left[∙\right]$ represents expectation, and *const* denotes a constant that is not dependent on the current variable. *Θ*\*Θ*_*k*_ represents the set of all latent variables except *Θ*_*k*_.

1) *Estimate latent variables G and H*: Combined with (3) and (4), the likelihood function $P\left(Y|G,H,W\right)$ contains exponential forms of *G* and *H*, resulting in it having no conjugate priors. Therefore, with reference to [45], we adopt the following approximation

$$\begin{array}{c} \sigma \left(z\right)\geq \sigma \left(\xi \right)exp\left\{\frac{z-\xi }{2}-\lambda \left(\xi \right)\left(z^{2}-\xi^{2}\right)\right\}\\ \lambda \left(\xi \right)=\frac{1}{2\xi }\left[\sigma \left(\xi \right)-\frac{1}{2}\right] \end{array}$$ (18)

where *σ*(*x*) = 1/(1 + *exp*(−*x*)) represents the sigmoid function. Combining (3), (4) and (18), the logarithmic likelihood of $Y_{ijk}$ satisfies (see S2 Text of S1 File for details)

$$\begin{array}{c} Ln\left[P\left(Y_{ijk}|G,H,W\right)\right]=Ln\left[{P_{ijk}}^{cY_{ijk}}{\left(1-P_{ijk}\right)}^{1-Y_{ijk}}\right]\\ \geq Ln\left(h(\xi_{ijk},G,H,W,)\right)=\left(\frac{cY_{ijk}-1+Y_{ijk}}{2}\right){\tilde{Y}}_{ijk}\\ -\left(cY_{ijk}+1-Y_{ijk}\right)\lambda \left(\xi_{ijk}\right){{\tilde{Y}}_{ijk}}^{2}+\left(c\xi_{ijk}+1-\xi_{ijk}\right)\\ Ln\left[\sigma \left(\xi_{ijk}\right)\right]-\left(cY_{ijk}+1-Y_{ijk}\right)\\ \frac{\xi_{ijk}}{2}+\left(cY_{ijk}+1-Y_{ijk}\right)\lambda \left(\xi_{ijk}\right){\xi_{ijk}}^{2} \end{array}$$ (19)

where *ξ*_*ijk*_ represents the local variation parameter. From (19), *Ln*(*h*(*ξ*_*ijk*_, *G*, *H*, *W*)) is quadratic functions of *G* and *H*, which is the lower bounds of log likelihood. Replace $P\left(Y|G,H,W\right)$ in (12) with *h*(*ξ*, *G*, *H*, *W*), combine (5), and substitute them into (17), it is found that the approximate posterior density of the *i*th row *G*_*i*∙_ of *G* obeys the multivariate Gaussian distribution with expectation ${\tilde{G}}_{i∙}$ and covariance matrix Σ(*G*_*i*∙_), that is, $q\left(G_{i∙}\right)=N\left(G_{i∙}|{\tilde{G}}_{i∙},\Sigma \left(G_{i∙}\right)\right)$ (see S3 Text of S1 File for details)

$$\begin{array}{c} \Sigma \left(G_{i\cdot }\right)={\left(2\overset{J}{\underset{j=1}{\sum }}\overset{K}{\underset{k=1}{\sum }}B_{ijk}\left[\left(\tilde{{H_{j\cdot }}^{T}H_{j\cdot }}\right)⊛\left(\tilde{{W_{k\cdot }}^{T}W_{k\cdot }}\right)\right]+{\tilde{\sigma }}_{g}E_{R}\right)}^{-1}\\ {\tilde{G}}_{i\cdot }={\left\{\Sigma \left(G_{i\cdot }\right)\left[{\left(\tilde{W}⊙\tilde{H}\right)}^{T}{A_{i\cdot }^{\left(1\right)}}^{T}+{\tilde{\sigma }}_{g}{\tilde{U}}^{T}{S_{i\cdot }^{u}}^{T}\right]\right\}}^{T} \end{array}$$ (20)

where $A_{ijk}=\frac{cY_{ijk}-1+Y_{ijk}}{2},\;B_{ijk}=(cY_{ijk}+1-Y_{ijk})\lambda \left(\xi_{ijk}\right)$. *A*^(1)^ represents the mode-1 matrixization of tensor A. ⊛ represents the Hadamard product, and ⊙ represents the Khatri-Rao product. *E*_*R*_ represents the identity matrix of size *R* × *R*. $\tilde{{H_{j∙}}^{T}H_{j∙}}$ and $\tilde{{W_{k∙}}^{T}W_{k∙}}$ denote the expectation of *H*_*j*∙_^*T*^*H*_*j*∙_ and *W*_*k*∙_^*T*^*W*_*k*∙_ respectively as follows:

$$\begin{array}{c} \tilde{{H_{j\cdot }}^{T}H_{j\cdot }}={{\tilde{H}}_{j\cdot }}^{T}{\tilde{H}}_{j\cdot }+\Sigma \left(H_{j\cdot }\right)\\ \tilde{{W_{k\cdot }}^{T}W_{k\cdot }}={{\tilde{W}}_{k\cdot }}^{T}{\tilde{W}}_{k\cdot }+\Sigma \left(W_{k\cdot }\right) \end{array}$$ (21)

Similarly, the posterior density of the *j*th row *H*_*j*∙_ of *H* obeys the multivariate Gaussian distribution with expectation ${\tilde{H}}_{j∙}$ and covariance matrix Σ(*H*_*j*∙_), that is, $q\left(H_{j∙}\right)=N\left(H_{j∙}|{\tilde{H}}_{j∙},\Sigma \left(H_{j∙}\right)\right)$

$$\begin{array}{c} \Sigma \left(H_{j\cdot }\right)={\left(2\overset{I}{\underset{i=1}{\sum }}\overset{K}{\underset{k=1}{\sum }}B_{ijk}\left[\left(\tilde{{W_{k\cdot }}^{T}W_{k\cdot }}\right)⊛\left(\tilde{{G_{i\cdot }}^{T}G_{i\cdot }}\right)\right]+{\tilde{\sigma }}_{h}E_{R}\right)}^{-1}\\ {\tilde{H}}_{j\cdot }={\left\{\Sigma \left(H_{j\cdot }\right)\left[{\left(\tilde{W}⊙\tilde{G}\right)}^{T}{A_{j\cdot }^{\left(2\right)}}^{T}+{\tilde{\sigma }}_{h}{\tilde{V}}^{T}{S_{j\cdot }^{d}}^{T}\right]\right\}}^{T} \end{array}$$ (22)

where **$\tilde{{G_{i∙}}^{T}G_{i∙}}={{\tilde{G}}_{i∙}}^{T}{\tilde{G}}_{i∙}+\Sigma \left(G_{i∙}\right),$** and *A*^(2)^ represents the mode-2 matrixization of tensor A.

2) *Estimate the latent variable W*: Similar to the solution of *G* and *H*, replace $P\left(Y_{ijk}|G,H,W\right)$ in (19) with *h*(*ξ*_*ijk*_, *G*, *H*, *W*), combine with (10), and substitute them into (17), it can be found that the approximate posterior density of the *k*th row *W*_*k*∙_ of *W* obeys the multivariate Gaussian distribution with expectation ${\tilde{W}}_{k∙}$ and covariance matrix Σ(*W*_*k*∙_), that is, $q\left(W_{k∙}\right)=N\left(W_{k∙}|{\tilde{W}}_{k∙},\Sigma \left(W_{k∙}\right)\right)$ (see S4 Text of S1 File for details)

$$\begin{array}{c} \Sigma \left(W_{k\cdot }\right)={\left[2\overset{I}{\underset{i=1}{\sum }}\overset{J}{\underset{j=1}{\sum }}B_{ijk}\left[\left(\tilde{{G_{i\cdot }}^{T}G_{i\cdot }}\right)⊛\left(\tilde{{H_{j\cdot }}^{T}H_{j\cdot }}\right)\right]+diag\left(\tilde{\lambda }\right)\right]}^{-1}\\ \tilde{W_{k\cdot }}={\left(\Sigma \left(W_{k\cdot }\right){\left(\tilde{H}⊙\tilde{G}\right)}^{T}{A_{k\cdot }^{\left(3\right)}}^{T}\right)}^{T} \end{array}$$ (23)

where *diag*(**∙**) represents the conversion of a vector to diagonal matrix form, and *A*^(3)^ represents the mode-3 matrixization of tensor A.

3) *Estimate latent variables U and V*: Substituting the priors of *U* and *G* into (17), the logarithmic posterior approximation of the *r*th column *U*_∙*r*_ of *U* satisfies (see S5 Text of S1 File for details)

$$\begin{array}{c} Lnq\left(U_{.r}\right)=E_{q\left(\Theta \backslash U_{.r}\right)}\left[Ln\left\{P\left(G|U,S^{m},\sigma_{g}\right)P\left(U|\lambda \right)\right\}\right]+const=\\ E_{q\left(\Theta \backslash U_{.r}\right)}\left[-\frac{{U_{.r}}^{T}\left[\sigma_{g}{S^{m}}^{T}S^{m}+\lambda_{r}E_{I}\right]U_{.r}-2\sigma_{g}{U_{.r}}^{T}{S^{m}}^{T}G_{\cdot r}}{2}\right]+const=\\ -\frac{{U_{.r}}^{T}\left[{\tilde{\sigma }}_{g}{S^{m}}^{T}S^{m}+{\tilde{\lambda }}_{r}E_{I}\right]U_{.r}-2{\tilde{\sigma }}_{g}{U_{.r}}^{T}{S^{m}}^{T}{\tilde{G}}_{\cdot r}}{2}+const \end{array}$$ (24)

From (24), the posterior approximation *U*_.*r*_ obeys the multivariate Gaussian distribution with expectation ${\tilde{U}}_{.r}$ and covariance matrix Σ(*U*_.*r*_), that is, $q\left(U_{.r}\right)=N\left(U_{.r}|{\tilde{U}}_{.r},\Sigma \left(U_{.r}\right)\right)$

$$\begin{array}{c} \Sigma \left(U_{.r}\right)={\left[{\tilde{\sigma }}_{g}{S^{m}}^{T}S^{m}+{\tilde{\lambda }}_{r}E_{I}\right]}^{-1}\\ {\tilde{U}}_{.r}={\tilde{\sigma }}_{g}\Sigma \left(U_{.r}\right){S^{u}}^{T}{\tilde{G}}_{\cdot r} \end{array}$$ (25)

Apparently, the posterior approximation *V*_.*r*_ also obeys the multivariate Gaussian distribution $q\left(V_{.r}\right)=N\left(V_{.r}|{\tilde{V}}_{.r},\Sigma \left({\tilde{V}}_{.r}\right)\right)$, as follows:

$$\begin{array}{c} \Sigma \left(V_{.r}\right)={\left[\sigma_{h}{S^{v}}^{T}S^{v}+\lambda_{r}E_{J}\right]}^{-1}\\ {\tilde{V}}_{.r}=\sigma_{h}\Sigma \left(V_{.r}\right){S^{v}}^{T}{\tilde{H}}_{\cdot r} \end{array}$$ (26)

4) *Estimate the latent variable* ***λ***: Substituting the priors of *U*, *V*, *W* and ***λ*** in (8), (9), (10) and (11) into (17), the logarithmic posterior approximately of ***λ*** satisfies (see S6 Text of S1 File for details)

$$\begin{array}{c} Lnq\left(\lambda \right)=E_{q\left(\Theta \backslash \lambda \right)}\left[Ln\left\{P\left(U|\lambda \right)P\left(V|\lambda \right)P\left(W|\lambda \right)P\left(\lambda |\alpha ,\beta \right)\right\}\right]\\ +const=\;\sum_{r=1}^{R}[\left(\frac{\left(I+J+K\right)}{2}+\alpha -1\right)Ln\left(\lambda_{r}\right)\\ -\left(\frac{\tilde{{U_{.r}}^{T}U_{.r}}+\tilde{{V_{.r}}^{T}V_{.r}}+\tilde{{W_{.r}}^{T}W_{.r}}}{2}+\beta \right)\lambda_{r}]+const \end{array}$$ (27)

From (27), the approximate posterior of *λ*_*r*_ follows a Gamma distribution with parameters ${\tilde{\alpha }}_{r}$ and ${\tilde{\beta }}_{r}$, that is, $q\left(\lambda_{r}\right)=Gamma\left(\lambda_{r}|{\tilde{\alpha }}_{r},{\tilde{\beta }}_{r}\right)$

$$\begin{array}{c} {\tilde{\alpha }}_{r}=\frac{\left(I+J+K\right)}{2}+\alpha \\ {\tilde{\beta }}_{r}=\left(\frac{\tilde{{U_{.r}}^{T}U_{.r}}+\tilde{{V_{.r}}^{T}V_{.r}}+\tilde{{W_{.r}}^{T}W_{.r}}}{2}+\beta \right)\\ {\tilde{\lambda }}_{r}=\frac{{\tilde{\alpha }}_{r}}{{\tilde{\beta }}_{r}}=\frac{I+J+K+2\alpha }{\tilde{{U_{.r}}^{T}U_{.r}}+\tilde{{V_{.r}}^{T}V_{.r}}+\tilde{{W_{.r}}^{T}W_{.r}}+2\beta } \end{array}$$ (28)

where ${\tilde{\lambda }}_{r}$ represents the expectation of *λ*_*r*_. In (28), $\tilde{{U_{.r}}^{T}U_{.r}},\;\tilde{{V_{.r}}^{T}V_{.r}}$, and $\tilde{{W_{.r}}^{T}W_{.r}}$ are the expectations of *U*_.*r*_^*T*^*U*_.*r*_, *V*_.*r*_^*T*^*V*_.*r*_, and *W*_.*r*_^*T*^*W*_.*r*_, respectively, as follows:

$$\begin{array}{c} \tilde{{U_{.r}}^{T}U_{.r}}={{\tilde{U}}_{\cdot r}}^{T}{\tilde{U}}_{\cdot r}+tr\left(\Sigma \left(U_{\cdot r}\right)\right)\\ \tilde{{V_{.r}}^{T}V_{.r}}={{\tilde{V}}_{\cdot r}}^{T}{\tilde{V}}_{\cdot r}+tr\left(\Sigma \left(V_{\cdot r}\right)\right)\\ \tilde{{W_{.r}}^{T}W_{.r}}={{\tilde{W}}_{.r}}^{T}{\tilde{W}}_{.r}+\sum_{k=1}^{K}\sigma^{2}\left(W_{kr}\right) \end{array}$$ (29)

where *tr*(∙) represents the trace of the square matrix, and *σ*^2^(∙) represents the variance.

5) *Estimate latent variables* *σ*_*g*_ *and σ*_*h*_: Substituting (5) and (7) into (17), the logarithmic posterior approximately of *σ*_*g*_ satisfies

$$\begin{array}{c} ln\;q\left(\sigma_{g}\right)=E\left[ln\left\{P\left(G|U,S^{u},\sigma_{g}\right)P\left(\sigma_{g}\right)\right\}\right]+const=\\ \left(\frac{IR}{2}-1\right)ln\left(\sigma_{g}\right)-\sigma_{g}\left(\frac{E\left[{\left\|G-S^{m}U\right\|}^{2}\right]}{2}\right)+const \end{array}$$ (30)

Therefore, the posterior distribution of *σ*_*g*_ is a Gamma distribution with mean

$${\tilde{\sigma }}_{g}=\frac{{\tilde{a}}^{g}}{{\tilde{b}}^{g}}=\frac{IR}{E\left[{\left\|G-S^{m}U\right\|}^{2}\right]}$$ (31)

Referring to Theorem 1 (see S7 Text of S1 File for details), $\left[{\left\|G-S^{m}U\right\|}^{2}\right]$ is transformed into

$$E\left[{\left\|G-S^{m}U\right\|}^{2}\right]={\left\|\tilde{G}-S^{m}\tilde{U}\right\|}^{2}+\sum_{i=1}^{I}tr\left(\Sigma \left(G_{i\cdot }\right)\right)+tr\left(S^{m}\sum_{r=1}^{R}\Sigma \left(U_{\cdot r}\right){\left(S^{m}\right)}^{T}\right)$$ (32)

Similarly, the posterior approximation of *σ*_*h*_ follows the Gamma distribution, whose expectation is

$${\tilde{\sigma }}_{h}=\frac{{\tilde{a}}^{h}}{{\tilde{b}}^{h}}=\frac{JR}{E\left[{\left\|H-S^{d}V\right\|}^{2}\right]}$$ (33)

6) *Estimate the local variation parameter ξ*_*ijk*_: From (19), take the derivative of *Ln*(*h*(*ξ*_*ijk*_, *G*, *H*, *W*)) with respect to *ξ*_*ijk*_, set the derivative equal to 0, and obtain *ξ*_*ijk*_ that satisfies (see S8 Text of S1 File for details)

$${\xi_{ijk}}^{2}=E\left({{\tilde{Y}}_{ijk}}^{2}\right)=E\left({\langle G_{i\cdot },H_{j\cdot },W_{k\cdot }\rangle }^{2}\right)=\langle \tilde{{U_{i\cdot }}^{T}U_{i\cdot }},\tilde{{V_{j\cdot }}^{T}V_{j\cdot }},\tilde{{W_{k\cdot }}^{T}W_{k\cdot }}\rangle$$ (34)

where 〈∙〉 is the generalized inner product, which means the product of corresponding elements, and then summed [42]. In (34), $\tilde{{U_{i∙}}^{T}U_{i∙}},\;\tilde{{V_{j∙}}^{T}V_{j∙}}$, and $\tilde{{W_{k∙}}^{T}W_{k∙}}$ are the expectations of ${U_{i∙}}^{T}U_{i∙},\;{V_{j∙}}^{T}V_{j∙}$, and ${W_{k∙}}^{T}W_{k∙}$, respectively, as follows:

$$\begin{array}{c} \tilde{{U_{i\cdot }}^{T}U_{i\cdot }}={{\tilde{U}}_{i\cdot }}^{T}{\tilde{U}}_{i\cdot }+diag\left(\sigma^{2}\left(U_{i1}\right),\sigma^{2}\left(U_{i2}\right),\cdots ,\sigma^{2}\left(U_{iR}\right)\right)\\ \tilde{{V_{j\cdot }}^{T}V_{j\cdot }}={{\tilde{V}}_{j\cdot }}^{T}{\tilde{V}}_{j\cdot }+diag\left(\sigma^{2}\left(V_{j1}\right),\sigma^{2}\left(V_{j2}\right),\cdots ,\sigma^{2}\left(V_{jR}\right)\right)\\ \tilde{{W_{k\cdot }}^{T}W_{k\cdot }}={{\tilde{W}}_{k\cdot }}^{T}{\tilde{W}}_{k\cdot }+\Sigma \left(W_{k\cdot }\right) \end{array}$$ (35)

In summary, the optimization algorithm for solving KBLTDARD is presented in Algorithm 1.

Algorithm 1. The Algorithm of KBLTDARD

Input: Known miRNA-disease-type tensor Y, miRNA similarity *S*^*m*^, disease similarity *S*^*d*^.

Output: *G*, *W*, *H*, and P.

Initialization:

 Initialize $\tilde{G},\;\tilde{H},\;\tilde{W},\;\tilde{U}$ and $\tilde{V}$ with multivariate Gaussian random numbers. Initialize Σ(*G*), Σ(*H*), Σ(*W*), Σ(*U*) and Σ(*V*) with unit tensors of corresponding size. Let local variational parameters *ξ*_*ijk*_ = 1, *i* = 1, ⋯, *I*, *j* = 1, ⋯, *J*, *k* = 1, ⋯, *K*.

repeat

 Update the posterior expectations ${\tilde{\sigma }}_{g}$ and ${\tilde{\sigma }}_{h}$ of *σ*_*g*_ and *σ*_*h*_ via (31) and (33), respectively.

 Update the posterior expectations and variances of *G*, *H* and *W* via (20), (22) and (23), respectively.

 Update the posterior expectations and variances of *U* and *V* via (25) and (26), respectively.

  for each *r* (1 ≤ *r* ≤ *R*)

   Update the posterior *q*(*λ*_*r*_) via (28) and (29).

  end for

 Update the local variational parameter *ξ* via (34) and (35).

Until convergence.

$P=\frac{1}{1+exp\left(-⟦G,W,H⟧\right)}$

   Return P.

Output: Factor matrices *G*, *W*, *H*, and association probability P.

### Complexity analysis

In algorithm 1, the time complexity is primarily attributed to the updating of the posterior expectation or variance of the latent variable and the iteration of the local variational parameters. Therefore, we combine the updated formula of latent variables to analyze one by one.

In (31) and (33), the computation cost of precision parameters *σ*_*g*_ and *σ*_*h*_ are $O\left(I^{3}R\right)$ and $O\left(J^{3}R\right)$ respectively. The computation cost of factor matrix *W* in (23) is $O\left(IJKR^{2}+KR^{3}\right)$, while the computation cost of factor matrix *G* in (20) and *H* in (22) are $O\left(IJKR^{2}+I^{2}R+IR^{3}\right)$ and $O\left(IJKR^{2}+J^{2}R+JR^{3}\right)$, respectively. The computation cost of latent variable *U* in (25) and *V* in (26) are $O\left(I^{3}R\right)$ and $O\left(J^{3}R\right)$, respectively. The computation cost of the rank determination parameter ***λ*** in (28) is $O\left(IR+JR+KR\right)$, while the computation cost of the local variational parameter *ξ* in (34) is $O\left(IJKR^{2}\right)$.

In summary, since the maximum number of iterations is fixed, the total computational cost of an update in Algorithm 1 is *O*(*IJKR*^2^ + *I*^3^*R* + *J*^3^*R* + *IR*^3^ + *JR*^3^ + *KR*^3^), where the potential space dimension *R* ≪ *min*{*I*, *J*}.

## Results

### Experimental settings

To extensively assess the predictive performance of multi-type miRNA-disease associations, referring to previous studies [22–25,27], we adopt two types of 5-fold cross validation.

1. *CV*_*type*_: Evaluation of the accuracy of model predictions for types. We randomly divided the miRNA-disease pairs with at least one type association into 5 disjoint equal parts. In each experiment, one subset was alternately selected as the test set and the rest as the training set.
2. *CV*_*triplet*_: Evaluation of the accuracy of model predictions for triples. We randomly divided all miRNA-disease--type triples into 5 disjoint equal parts. In each experiment, one subset was alternately selected as the test set and the rest as the training set.

Regarding *CV*_*type*_, we are interested in the type with the maximum score in the test set. Therefore, referring to previous studies [22–25,27,41], we sorted the types of miRNA-disease pairs according to prediction scores, selected the type with the highest score, and applied the average Top-1 precision, average Top-1 recall, and average Top-1 F1 as evaluation indicators. For *CV*_*triplet*_, we choose commonly used overall evaluation indicators, namely the area under the precision-recall curve (AUPR), the area under the ROC curve (AUC), and the F1 value [22,24,27]. In this study, the Matlab tensor toolbox "tensor_toolbox" is used to perform tensor calculations [46].

### Comparison experiments

Several computational models have been developed and applied for the prediction of multiple types of miRNA-disease associations. To comprehensively evaluate the performance of KBLTDARD, we select six state-of-the-art tensor decomposition models as benchmarks.

TFAI [22,47]: TFAI introduces graph Laplacian regularization based on the CP decomposition to keep the information about the local structure of the data.

FBCPARD [42]: FBCPARD is a standard Bayesian tensor decomposition model, which introduces the Bayesian framework into CP decomposition and utilizes automatic rank determination to achieve adaptive inference of CP rank.

TDRC [22]: TDRC established a new way of relation constraint and integrated auxiliary information of miRNAs and diseases into CP decomposition.

WeightTDAIGN [27]: WeightTDAIGN introduces a positive sample weighting strategy based on CP decomposition to improve prediction performance, utilizes the *L*_2,1_ norm constraint projection matrix to reduce the impact of redundant information, and employs graph regularization to preserve local structural information.

TFLP [25]: TFLP combines tensor robust principal component analysis and label propagation, introduces multiple similarity information of miRNAs (diseases), and achieves prediction through iteration of label information.

SPLDHyperAWNTF [24]: SPLDHyperAWNTF integrates hypergraph learning and tensor weighting with non-negative tensor decomposition to achieve miRNA disease-type triple prediction.

Except for FBCPARD, the above five methods are applied to multiple types of miRNA-disease association prediction. Therefore, we adopt the optimal parameters recommended by the above methods to perform experiments. In the original literature, WeightTDAIGN, TFLP, SPLDHyperAWNTFTDRC, and TFAI all construct miRNA functional similarity through miRNA-disease associations, which results in bias towards known miRNA-disease associations. Therefore, different from the original method, this paper adopts the functional similarity of miRNAs described in Section 2.2.2 to perform the above model. In addition, when performing *CV*_*triplet*_, the random selection of negative samples may have an impact on the evaluation metrics of the model. Therefore, referring to the suggestion of Huang et al. [22], for each experiment, we perform negative sample selection under 20 different seeds and calculate the mean as the final evaluation index.

According to Table 1, under the *CV*_*type*_, KBLTDARD outperforms other methods on both HMDD v2.0 and HMDD v3.2. Specifically, on HMDD v2.0, KBLTDARD’s Top-1 precision, Top-1 recall and Top-1 F1 reached 0.6361, 0.5665 and 0.5869 respectively, which are 6.87%, 6.79% and 6.57% higher than the second-ranked SPLDHyperAWNTF. On HMDD v3.2, the Top-1 precision, Top-1 recall and Top-1 F1 of KBLTDARD are 0.6236, 0.4783 and 0.5197, respectively, which are substantially better than other methods.

**Table 1:** The predictive performance of all models under *CV*_*type*_.

Method	HMDD v2.0			HMDD v3.2		
	Top-1 precision	Top-1 recall	Top-1 F1	Top-1 precision	Top-1 recall	Top-1 F1
TFAI	0.5530	0.4928	0.5078	0.5827	0.4470	0.4801
FBCPARD	0.5349	0.4765	0.4903	0.5387	0.4132	0.4445
TDRC	0.5195	0.463	0.4727	0.5472	0.4201	0.4455
WeightTDAIGN	0.5134	0.4571	0.4697	0.5716	0.4385	0.4714
TFLP	0.3659	0.3260	0.3334	0.3133	0.2402	0.2488
SPLDHyperAWNTF	0.5952	0.5305	0.5507	0.6056	0.4647	0.5024
KBLTDARD	**0.6361**	**0.5665**	**0.5869**	**0.6236**	**0.4783**	**0.5197**

Note: Bold numbers indicate the optimal value under the corresponding indicator.

According to Table 2, under the *CV*_*triplet*_, KBLTDARD also performs better prediction performance on HMDD v2.0 and HMDD v3.2. Specifically, on HMDD v2.0, the AUPR of KBLTDARD is 0.8966, which is 5.00%, 16.29%, 4.23%, 9.74%, 9.08% and 1.69% higher than that of TFAI (0.8539), FBCPARD (0.7710), TDRC (0.8602), WeightTDAIGN (0.8170), TFLP (0.8220) and SPLDHyperAWNTF (0.8817), respectively. The AUC and F1 of HMDD v2.0 are 0.8893 and 0.8218 respectively, which are substantially better than other methods. Furthermore, on HMDD v3.2, the AUPR, AUC and F1 of KBLTDARD are 0.9452, 0.9445 and 0.8775 respectively, which is better than other methods. The comparison of KBLTDARD with other models under 20 random seeds is detailed in S9 Text of S1 File.

**Table 2:** The predictive performance of all models under *CV*_*triplet*_.

Method	HMDD v2.0			HMDD v3.2		
	AUPR	AUC	F1	AUPR	AUC	F1
TFAI	0.8539	0.8194	0.7742	0.9211	0.9046	0.8498
FBCPARD	0.7710	0.6764	0.6746	0.8741	0.8239	0.8027
TDRC	0.8602	0.8264	0.7754	0.9243	0.9121	0.8517
WeightTDAIGN	0.8170	0.7434	0.7229	0.9099	0.8841	0.8405
TFLP	0.8220	0.7639	0.7771	0.8193	0.7447	0.7774
SPLDHyperAWNTF	0.8817	0.8646	0.8025	0.9294	0.9214	0.8569
KBLTDARD	**0.8966**	**0.8835**	**0.8185**	**0.9452**	**0.9445**	**0.8775**

Note: Bold numbers indicate the optimal value under the corresponding indicator.

In summary, KBLTDARD achieved the most optimal prediction performance, followed by SPLDHyperAWNTF. FBCPARD’s prediction ability was limited to some extent as it solely relied on miRNA-disease-type associations for prediction. Furthermore, models other than KBLTDARD contain many hyperparameters that often demand cumbersome debugging before conducting predictions, greatly affecting their computational efficiency and generalization capabilities. In contrast, KBLTDARD, with the help of Bayesian framework, takes low-dimensional features and model hyperparameters as latent variables, and realizes model solution by inferring the posterior distribution of latent variables, avoiding complex parameter debugging and enhancing generalization ability.

### Ablation studies

Compared with the traditional Bayesian tensor decomposition methods [42,44], the improvement of KBLTDARD is manifested in three aspects: the introduction of logical functions, the addition of auxiliary information, and the set of importance levels. For a better understanding of these contributions, we created comparison models by removing the logistic function, auxiliary information, and importance level from KBLTDARD, respectively. Specifically, KBTD represents the model acquired by removing the logistic function from KBLTDARD, BLTD represents the model acquired by removing the auxiliary information from KBLTDARD, and KBLTD-NOC represents the model acquired by eliminating the importance level from KBLTDARD.

**Fig 3:**
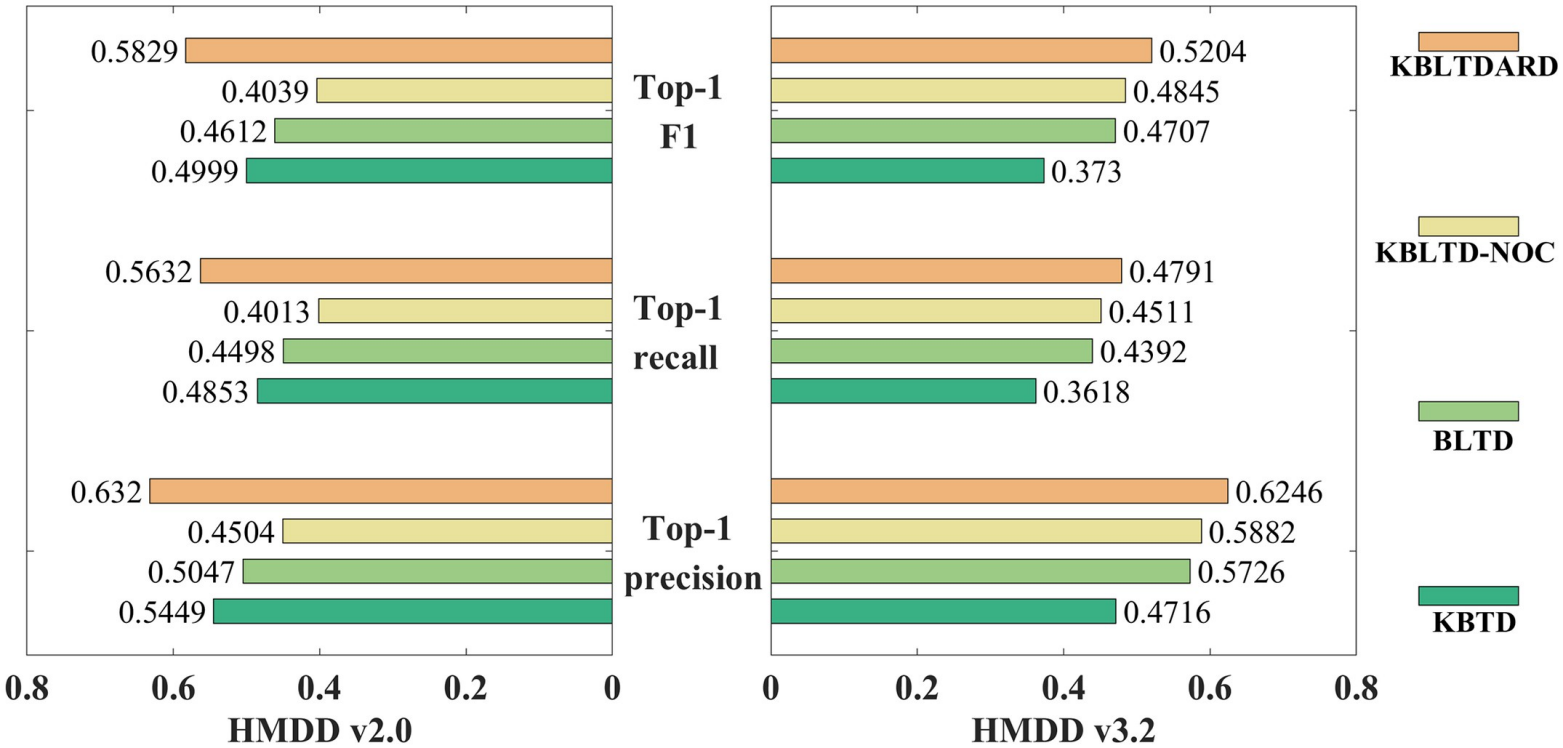
The prediction performance of ablation experiment.

Fig 3 shows the prediction performance of ablation experiments evaluated by 5-fold cross-validation under HMDD v2.0 and HMDD v3.2. For KBLTD-NOC and KBLTDARD, after setting the importance level, the predictive ability of KBLTDARD is substantially superior to that of KBLTD-NOC, especially on the sparse data set (HMDD v2.0). This result indicates that increasing the importance of known associations can effectively mitigate the influence of false-negative samples on model performance. For BLTD and KBLTDARD, after combining auxiliary information, KBLTDARD achieves higher prediction performance compared with BLTD. This result shows that the introduction of auxiliary information corrects the iteration direction of logical tensor decomposition and improves the model’s prediction ability for isolated samples. For KBTD and KBLTDARD, after the introduction of logistic functions, the prediction performance of KBLTDARD has been significantly improved. The result of this experiment demonstrates that the introduction of logical functions significantly improves nonlinear learning capabilities. To sum up, the addition of logical functions, auxiliary information, and importance levels can improve the predictive ability of the model to a certain extent.

### Case study

To further evaluate the actual prediction performance of KBLTDARD, we conduct two types of case studies. The first strategy evaluates KBLTDARD from a global perspective, that is, testing the model’s predictive ability for all diseases. Therefore, we employ KBLTDARD to predict 447 diseases in HMDD v3.2 one by one. The second strategy predicts four common diseases (‘Gastric Neoplasms’, ‘Myocardial Infarction’, ‘Prostate Neoplasms’ and ‘Pancreatic Neoplasms’) and checks the latest literature to test the prediction results.

In Case Study 1, for each disease from HMDD v3.2, all its associations with all miRNAs and types are removed, and the prediction score for each disease is evaluated. We adopt AUC to evaluate the overall predictive performance with respect to each disease and perform statistics on all AUC values. In addition, for each disease, researchers are more likely to focus on the fraction of known associations included in the top-ranked associations [38,48], that is, the hit rate, as follows:

$$\text{Hitrate}\left(\rho \right)=\frac{\left|S_{cand}\left(\left[\rho \cdot N\right]\right)\cap S_{Test}\right|}{\left|S_{Test}\right|}$$ (36)

where *N* is the total number of triples in the test set, *ρ* represents the scaling factor, which in this study is selected as {1%, 5%, 10%}, and [∙] indicates an integer operation. *S*_*cand*_ ([*ρ* ∙ *N*]) denotes the set of highest scoring top [*ρ* ∙ *N*] triples, and *S*_*Test*_ denotes the real triple set in the test set.

**Fig 4:**
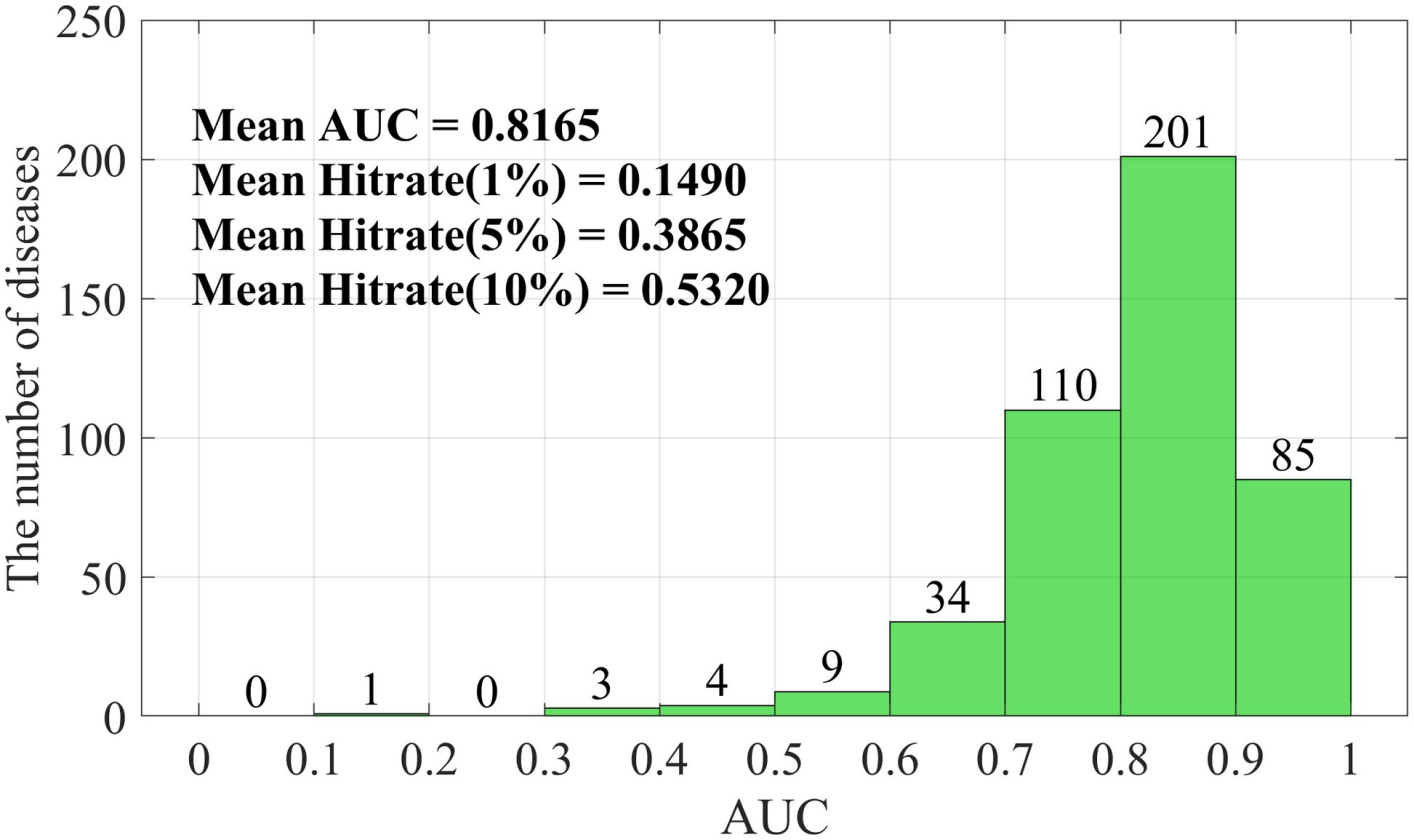
The prediction results of KBLTDARD for all diseases.

As presented in Fig 4, the average AUC of KBLTDARD is 0.8165. Among 447 diseases predicted by KBLTDARD, the AUC of 286 diseases exceeded 0.8, amounting to 63.98%. Only 17 diseases had AUC less than 0.6, amounting to less than 4%. The average hit rates of the top 1%, top 5%, and top 10% are 0.1490, 0.3865, and 0.5320 respectively, which are 14 times, 7 times, and 5 times more than the random hit rates (0.01, 0.05, and 0.10) respectively. The above results indicate that the associations with top prediction scores contain the vast majority of known associations.

Then we focus on these four common diseases: Gastric Neoplasms, Myocardial Infarction, Prostate Neoplasms and Pancreatic Neoplasms for further analysis. Table 3 shows the top 20 predictions and related evidence for the disease Gastric Neoplasms, and S1, S2, and S3 Tables show the top 20 predictions and related evidence for the three diseases Myocardial Infarction, Prostate Neoplasms, and Pancreatic Neoplasms, respectively.

**Table 3:** Gastric Neoplasms-related miRNAs and types predicted by KBLTDARD.

Rank	MiRNA	Type	PMID	Rank	MiRNA	Type	PMID
1	hsa-mir-146a	tissue	20726036	11	hsa-mir-206	target	23348698
2	hsa-mir-21	target	21081469	12	hsa-mir-122	tissue	22112324
3	hsa-mir-21	tissue	16461460	13	hsa-mir-223	tissue	16461460
4	hsa-mir-155	tissue	22112324	14	hsa-mir-210	tissue	Unconfirmed
5	hsa-mir-29a	tissue	25889078	15	hsa-mir-483	target	Unconfirmed
6	hsa-mir-146a	target	22020746	16	hsa-mir-483	circulation	Unconfirmed
7	hsa-mir-21	circulation	22860003	17	hsa-mir-146a	circulation	23806809
8	hsa-mir-206	tissue	22112324	18	hsa-mir-125a	tissue	21987613
9	hsa-mir-155	target	26056431	19	hsa-mir-21	genetics	25230738
10	hsa-mir-146a	genetics	21632853	20	hsa-mir-34b	tissue	19148490

Gastric cancer is the fifth most prevalent cancer in the world and the third major cause of cancer-related deaths worldwide [49]. There are more than 1 million new cases of gastric cancer worldwide every year, and the number of gastric cancer-related deaths exceeds 780,000 [50]. Tchernitsa et al. [51] studied the differential expression of miRNA in adjacent normal and tumor samples from gastric cancer patients. The results found that miR-146a was significantly different in lymph node-positive and node-negative gastric cancer, and its changes may affect local tumor growth and lymph node spread. Zhang et al. [52] found that hsa-mir-21 is up-regulated in gastric cancer tissues and is significantly related to the degree of differentiation, local invasion and lymph node metastasis of tumor tissues. As shown in Table 3, among the top 20 miRNA and type associations predicted by KBLTDARD, 17 have been verified by relevant literature. Furthermore, in S1, S2,and S3 Tables, among the top 20 associations predicted by KBLTDARD for Myocardial Infarction, Prostate Neoplasms, and Pancreatic Neoplasms, 16, 16, and 17 were confirmed, respectively.

## Discussion and conclusion

In this paper, we proposed a new KBLTDARD model to predict higher-order relationships between miRNAs, diseases and types. This model utilizes miRNA-gene association and gene similarity to calculate the functional similarity of miRNAs, avoiding the re-use of known miRNA-disease associations. Then, we combine logistic tensor decomposition and Bayesian inference, introduce auxiliary information, and build a probabilistic graphical model to describe the dependence between latent variables. In addition, with regard to KBLTDARD, we developed an efficient deterministic Bayesian inference algorithm to ensure the efficiency of the model solution. Under the 5-CV framework, the top-1 precision of KBLTDARD for new type prediction reached 0.6320 and 0.6246, and the AUC values for new triplet prediction reached 0.8834 and 0.9445, respectively, on HMDD v2.0 and HMDD v3.2 datasets. The results show that the performance of KBLTDARD is significantly improved compared to the previous methods. Case studies of ’gastric neoplasia’, ’myocardial infarction’, ’prostate neoplasia’ and ’pancreatic neoplasia’ also demonstrated the predictive power of KBLTDARD. Taken together, these results suggest that KBLTDARD can effectively observe multiple types of miRNA-disease associations.

It should be noted that the following factors can contribute to the reliable performance of KBLTDARD. First, we extract important information from the related database of miRNA and disease, and mine key features from the trained tensors to ensure the richness of information about miRNAs and diseases. In addition, we combined logical tensor decomposition and Bayesian inference to realise the automatic search of hyperparameters, which improves the nonlinear learning ability and generalisation ability of the model.

However, there are some limitations that may affect the performance of KBLTDARD. First, to facilitate model inference, we selected Gaussian and Gamma distributions with conjugation properties to represent prior distributions of latent variables, which may not be optimal for model representations. In future research, we will further explore Bayesian theory and try more advanced prior distributions. Second, although our model shows stronger learning ability than some deep learning, in future studies we will try to combine some advanced deep learning methods (such as hypergraph neural networks, etc.) to improve the nonlinear representation ability of the model.

## Supporting information

S1 File Supporting information. (DOCX)

S1 Fig The boxplot of prediction results of KBLTDARD under *CV*_*triplet*_. (TIF)

S1 Table Myocardial Infarction-related miRNAs and association types predicted by KBLTDARD. (DOCX)

S2 Table Prostate Neoplasms-related miRNAs and association types predicted by KBLTDARD. (DOCX)

S3 Table Pancreatic Neoplasms-related miRNAs and association types predicted by KBLTDARD. (DOCX)

S4 Table Statistics of predictions for all models under *CV*_*triplet*_. (DOCX)

S5 Table The P-value of the Wilcoxon rank sum test for pairing KBLTDARD with other models. (DOCX)
