# Supplementary material for: Kernel Bayesian logistic tensor decomposition with automatic rank determination for predicting multiple types of miRNA-disease associations
Source: PLoS Comput Biol. 2024 Jul 8;20(7):e1012287. doi: 10.1371/journal.pcbi.1012287 (PMC11257412; doi:10.1371/journal.pcbi.1012287)
Supplement: S1 File — (DOCX) [file pcbi.1012287.s001.docx]

# Kernel Bayesian logistic tensor decomposition with automatic rank determination for predicting multiple types of miRNA-disease associations

# S1 Text: The log of the joint distribution

The joint distribution is

| $q\left( \Theta\right)=\prod_{k} q\left( \Theta_{k} \right)=q(G)q(H)q(W)q(U)q(V)q(\boldsymbol{\lambda})q\left( \sigma_{g} \right)q\left( \sigma_{h} \right)$,  where $\Theta=\left\{ G,H,W,U,V,\boldsymbol{\lambda},\sigma_{g},\sigma_{h} \right\}$ | (1) |
| --- | --- |

Hence, the log of joint distribution can be written as

| $\mathcal{l}\left( \Theta\right)=\sum_{i=1}^{I} \sum_{j=1}^{J} \sum_{k=1}^{K} c\mathcal{Y}_{ijk}Ln\left( \sigma\left( {\tilde{\mathcal{Y}}}_{ijk} \right) \right)+\left( 1-\mathcal{Y}_{ijk} \right)Ln\left( 1-\sigma\left( {\tilde{\mathcal{Y}}}_{ijk} \right) \right)+\left( \frac{IRln\left( \sigma_{g} \right)}{2}-\frac{\left\Vert G-S^{m}U \right\Vert_{F}^{2}\sigma_{g}}{2} \right)+\left( \frac{JRln\left( \sigma_{h} \right)}{2}-\frac{\left\Vert H-S^{d}V \right\Vert_{F}^{2}\sigma_{h}}{2} \right)+\left( \frac{Ktr\left( ln\left( \Lambda\right) \right)}{2}-\frac{tr\left( W\Lambda W^{T} \right)}{2} \right)+\left( \frac{Itr\left( ln\left( \Lambda\right) \right)}{2}-\frac{tr\left( U\Lambda U^{T} \right)}{2} \right)+\left( \frac{Jtr\left( ln\left( \Lambda\right) \right)}{2}-\frac{tr\left( V\Lambda V^{T} \right)}{2} \right)+\left( \left( \alpha-1 \right)tr\left( ln\left( \Lambda\right) \right)-\beta tr\left( \Lambda\right) \right)-ln\left( \sigma_{g} \right)-ln\left( \sigma_{h} \right)+const=\sum_{i=1}^{I} \sum_{j=1}^{J} \sum_{k=1}^{K} c\mathcal{Y}_{ijk}Ln\left( \sigma\left( {\tilde{\mathcal{Y}}}_{ijk} \right) \right)+\left( 1-\mathcal{Y}_{ijk} \right)Ln\left( 1-\sigma\left( {\tilde{\mathcal{Y}}}_{ijk} \right) \right)-\frac{1}{2}\left[ \sigma_{g}\left\Vert G-S^{m}U \right\Vert_{F}^{2}+\sigma_{h}\left\Vert H-S^{d}V \right\Vert_{F}^{2} \right]-\frac{1}{2}\left[ tr\left( W\Lambda W^{T} \right)+tr\left( U\Lambda U^{T} \right)+tr\left( V\Lambda V^{T} \right)+2\beta tr\left( \Lambda\right) \right]+\left( \frac{IR}{2}-1 \right)ln\left( \sigma_{g} \right)+\left( \frac{JR}{2}-1 \right)ln\left( \sigma_{h} \right)+\left( \frac{I+J+K}{2}+\alpha-1 \right)tr\left( ln\left( \Lambda\right) \right)$ | (1) |
| --- | --- |

where $\Lambda=diag\left( \lambda_{1},\lambda_{2},\cdots,\lambda_{R} \right)$, $ln\left( \Lambda\right)=diag\left( ln\left( \lambda_{1} \right),ln\left( \lambda_{2} \right),\cdots,ln\left( \lambda_{R} \right) \right)$, and $diag\left( \boldsymbol{\cdot} \right)$ means converting the vector into a diagonal matrix, and $tr\left( \cdot\right)$ means calculating the trace of the square matrix. $const$ represents a constant that has nothing to do with $\Theta$, and $\mathcal{l}\left( \Theta\right)$ represents the logarithmic joint distribution, that is, $lnp(\Theta,\mathcal{Y})$. This form will be used frequently in variational Bayesian inference.

# S2 Text: The logarithmic likelihood of $\mathcal{Y}_{\boldsymbol{ijk}}$

| $Ln\left[ P\left( \mathcal{Y}_{ijk} \vert G,H,W \right) \right]=Ln\left[ {\mathcal{P}_{ijk}}^{c\mathcal{Y}_{ijk}}{(1-\mathcal{P}_{ijk})}^{1-\mathcal{Y}_{ijk}} \right]=c\mathcal{Y}_{ijk}Ln\left( \sigma\left( {\tilde{\mathcal{Y}}}_{ijk} \right) \right)+(1-\mathcal{Y}_{ijk})Ln\left( 1-\sigma\left( {\tilde{\mathcal{Y}}}_{ijk} \right) \right)=c\mathcal{Y}_{ijk}Ln\left( \frac{1}{1+exp(-{\tilde{\mathcal{Y}}}_{ijk})} \right)+(1-\mathcal{Y}_{ijk})Ln\left( 1-\frac{1}{1+exp(-{\tilde{\mathcal{Y}}}_{ijk})} \right)=c\mathcal{Y}_{ijk}{\tilde{\mathcal{Y}}}_{ijk}+(c\mathcal{Y}_{ijk}+1-\mathcal{Y}_{ijk})Ln\left( \sigma\left( -{\tilde{\mathcal{Y}}}_{ijk} \right) \right)\geq Ln\left( h\left（ \xi_{ijk},G,H,W \right） \right)=c\mathcal{Y}_{ijk}{\tilde{\mathcal{Y}}}_{ijk}+(c\mathcal{Y}_{ijk}+1-\mathcal{Y}_{ijk})\left\{ Ln\left[ \sigma\left( \xi_{ijk} \right) \right]-\frac{{\tilde{\mathcal{Y}}}_{ijk}+\xi_{ijk}}{2}-\lambda\left( \xi_{ijk} \right)\left( {{\tilde{\mathcal{Y}}}_{ijk}}^{2}-{\xi_{ijk}}^{2} \right) \right\}=\left( \frac{c\mathcal{Y}_{ijk}-1+\mathcal{Y}_{ijk}}{2} \right){\tilde{\mathcal{Y}}}_{ijk}-(c\mathcal{Y}_{ijk}+1-\mathcal{Y}_{ijk})\lambda\left( \xi_{ijk} \right){{\tilde{\mathcal{Y}}}_{ijk}}^{2}+\left( c\xi_{ijk}+1-\xi_{ijk} \right)Ln\left[ \sigma\left( \xi_{ijk} \right) \right]-(c\mathcal{Y}_{ijk}+1-\mathcal{Y}_{ijk})\frac{\xi_{ijk}}{2}+(c\mathcal{Y}_{ijk}+1-\mathcal{Y}_{ijk})\lambda\left( \xi_{ijk} \right){\xi_{ijk}}^{2}$ | (1) |
| --- | --- |

It should be noted that in the above equation, only the red part contains ${\tilde{\mathcal{Y}}}_{ijk}$, while the black part is a constant when calculating $G$, $H$, and $W$. Therefore, for the convenience of writing, let

| $\mathcal{A}_{ijk}=\frac{c\mathcal{Y}_{ijk}-1+\mathcal{Y}_{ijk}}{2}$  $\mathcal{B}_{ijk}=\left( c\mathcal{Y}_{ijk}+1-\mathcal{Y}_{ijk} \right)\lambda\left( \xi_{ijk} \right)$  $\mathcal{F}_{ijk}=\left( c\xi_{ijk}+1-\xi_{ijk} \right)Ln\left[ \sigma\left( \xi_{ijk} \right) \right]-(c\mathcal{Y}_{ijk}+1-\mathcal{Y}_{ijk})\frac{\xi_{ijk}}{2}+(c\mathcal{Y}_{ijk}+1-\mathcal{Y}_{ijk})\lambda\left( \xi_{ijk} \right){\xi_{ijk}}^{2}$ | (2) |
| --- | --- |

Then, equation (1) transforms into

| $Ln\left[ P\left( \mathcal{Y}_{ijk} \vert G,H,W \right) \right]\geq Ln\left( h\left（ \xi_{ijk},G,H,W \right） \right)=\mathcal{A}_{ijk}{\tilde{\mathcal{Y}}}_{ijk}-\mathcal{B}_{ijk}{{\tilde{\mathcal{Y}}}_{ijk}}^{2}+\mathcal{F}_{ijk}$ | (3) |
| --- | --- |

# S3 Text: The variational posterior distribution of $\boldsymbol{G}$

| $Lnq\left( G_{i\cdot} \right)=\mathbb{E}_{q\left( \Theta\backslash G_{i\cdot} \right)}\left[ Ln\left\{ P\left( \mathcal{Y} \vert G,H,W \right)P\left( G \vert U,S^{u},\sigma_{g} \right) \right\} \right]\geq\mathbb{E}_{q\left( \Theta\backslash G_{i\cdot} \right)}\left[ Ln\left\{ h\left（ \xi,G,H,W \right）P\left( G \vert U,S^{u},\sigma_{g} \right) \right\} \right]=\mathbb{E}_{q\left( \Theta\backslash G_{i\cdot} \right)}.\left\{ \sum_{j=1}^{J} \sum_{k=1}^{K} \left[ \mathcal{A}_{ijk}{\tilde{\mathcal{Y}}}_{ijk}-\mathcal{B}_{ijk}{{\bar{\mathcal{Y}}}_{ijk}}^{2} \right]-\frac{\sigma_{g}{\left( G_{i\cdot}-S_{i\cdot}^{u}U \right)\left( G_{i\cdot}-S_{i\cdot}^{u}U \right)}^{T}}{2} \right\}+const$ | (4) |
| --- | --- |

Then, ${\tilde{\mathcal{Y}}}_{ijk}$ and ${{\tilde{\mathcal{Y}}}_{ijk}}^{2}$ are transformed as follows：

| ${\tilde{\mathcal{Y}}}_{ijk}=\left\langle G_{i\cdot},H_{j\cdot},W_{k\cdot} \right\rangle=\sum_{r=1}^{R} G_{ir}H_{jr}W_{kr}=G_{i\cdot}\left( H_{j\cdot}⊛W_{k\cdot} \right)^{T}$  ${{\tilde{\mathcal{Y}}}_{ijk}}^{2}=G_{i\cdot}\left( H_{j\cdot}⊛W_{k\cdot} \right)^{T}\left( H_{j\cdot}⊛W_{k\cdot} \right){G_{i\cdot}}^{T}=G_{i\cdot}\left( \left( {H_{j\cdot}}^{T}H_{j\cdot} \right)⊛\left( {W_{k\cdot}}^{T}W_{k\cdot} \right) \right){G_{i\cdot}}^{T}$ | (5) |
| --- | --- |

Therefore, $\sum_{j=1}^{J} \sum_{k=1}^{K} \mathcal{A}_{ijk}{\tilde{\mathcal{Y}}}_{ijk}$ is calculated as follows:

| $\sum_{j=1}^{J} \sum_{k=1}^{K} \mathcal{A}_{ijk}{\tilde{\mathcal{Y}}}_{ijk}=G_{i\cdot}\sum_{j=1}^{J} \sum_{k=1}^{K} \mathcal{A}_{ijk}\left( H_{j\cdot}⊛W_{k\cdot} \right)^{T}=G_{i\cdot}{\left( W\odot H \right)^{T}\mathcal{A}_{i\cdot}^{(1)}}^{T}$ | (6) |
| --- | --- |

Substituting (5) and (6) into equation (4), we get

| $\mathbb{E}_{q\left( \Theta\backslash G_{i\cdot} \right)}\left[ Ln\left( P\left( \mathcal{Y} \vert G,H,W \right)P\left( G \vert U,S^{u},\sigma_{g} \right) \right) \right]\mathbb{\geq E}\left[ G_{i\cdot}{\left( W\odot H \right)^{T}\mathcal{A}_{i\cdot}^{(1)}}^{T}-G_{i\cdot}\left( \sum_{j=1}^{J} \sum_{k=1}^{K} \mathcal{B}_{ijk}\left[ \left( {H_{j\cdot}}^{T}H_{j\cdot} \right)⊛\left( {W_{k\cdot}}^{T}W_{k\cdot} \right) \right] \right){G_{i\cdot}}^{T}-\frac{\sigma_{g}{\left( G_{i\cdot}-S_{i\cdot}^{u}U \right)\left( G_{i\cdot}-S_{i\cdot}^{u}U \right)}^{T}}{2} \right]+const\mathbb{=E}\left[ -\frac{G_{i\cdot}\left( 2\sum_{j=1}^{J} \sum_{k=1}^{K} \mathcal{B}_{ijk}\left[ \left( {H_{j\cdot}}^{T}H_{j\cdot} \right)⊛\left( {W_{k\cdot}}^{T}W_{k\cdot} \right) \right]+\sigma_{g}E_{R} \right){G_{i\cdot}}^{T}}{2}+G_{i\cdot}\left( {\left( W\odot H \right)^{T}\mathcal{A}_{i\cdot}^{(1)}}^{T}+\sigma_{g}U^{T}{S_{i\cdot}^{u}}^{T} \right) \right]+const$ | (7) |
| --- | --- |

Therefore, the posterior approximation of $G_{i\cdot}$ satisfies the multivariate Gaussian distribution, and its posterior parameters are obtained in the form of the above Gaussian distribution.

# S4 Text: The variational posterior distribution of $\boldsymbol{W}$

| $Lnq\left( W_{k\cdot} \right)=\mathbb{E}_{q\left( W_{k\cdot} \right)}\left[ Ln\left\{ P\left( \mathcal{Y} \vert G,H,W \right)P\left( W \vert\lambda\right) \right\} \right]\geq\mathbb{E}_{q\left( W_{k\cdot} \right)}\left[ Ln\left\{ h\left（ \xi,G,H,W \right）P\left( W \vert\lambda\right) \right\} \right]=\mathbb{E}_{q\left( \Theta\backslash W_{k\cdot} \right)}.\left\{ \sum_{ij} \left[ \mathcal{A}_{ijk}{\tilde{\mathcal{Y}}}_{ijk}-\mathcal{B}_{ijk}{{\tilde{\mathcal{Y}}}_{ijk}}^{2} \right]+\sum_{r=1}^{R} \left( -\frac{\lambda_{r}\left( W_{k,r} \right)^{2}}{2} \right) \right\}+const$ | (8) |
| --- | --- |

Then, ${\tilde{\mathcal{Y}}}_{ijk}$ and ${{\tilde{\mathcal{Y}}}_{ijk}}^{2}$ are transformed as follows：

| ${\tilde{\mathcal{Y}}}_{ijk}=\left\langle G_{i\cdot},H_{j\cdot},W_{k\cdot} \right\rangle=\sum_{r=1}^{R} G_{ir}H_{jr}W_{kr}=W_{k\cdot}\left( G_{i\cdot}⊛H_{j\cdot} \right)^{T}$  ${{\tilde{\mathcal{Y}}}_{ijk}}^{2}=W_{k\cdot}\left( G_{i\cdot}⊛H_{j\cdot} \right)^{T}\left( G_{i\cdot}⊛H_{j\cdot} \right){W_{k\cdot}}^{T}=W_{k\cdot}\left( \left( {G_{i\cdot}}^{T}G_{i\cdot} \right)⊛\left( {H_{j\cdot}}^{T}H_{j\cdot} \right) \right){W_{k\cdot}}^{T}$ | (9) |
| --- | --- |

Therefore, $\sum_{j=1}^{J} \sum_{k=1}^{K} \mathcal{A}_{ijk}{\tilde{\mathcal{Y}}}_{ijk}$ is calculated as follows:

| $\sum_{i=1}^{I} \sum_{j=1}^{J} \mathcal{A}_{ijk}{\tilde{\mathcal{Y}}}_{ijk}=W_{k\cdot}\sum_{i=1}^{I} \sum_{j=1}^{J} \mathcal{A}_{ijk}\left( G_{i\cdot}⊛H_{j\cdot} \right)^{T}=W_{k\cdot}{\left( H\odot G \right)^{T}\mathcal{A}_{k\cdot}^{(3)}}^{T}$ | (10) |
| --- | --- |

Substituting (9) and (10) into equation (8), we get

| $Lnq\left( W_{k\cdot} \right)\geq\mathbb{E}_{q\left( \Theta\backslash W_{k\cdot} \right)}\left\{ \sum_{i=1}^{I} \sum_{j=1}^{J} \left[ \mathcal{A}_{ijk}{\tilde{\mathcal{Y}}}_{ijk}-\mathcal{B}_{ijk}{{\tilde{\mathcal{Y}}}_{ijk}}^{2} \right]+\sum_{r=1}^{R} \left( -\frac{\lambda_{r}\left( W_{k,r} \right)^{2}}{2} \right) \right\}+const\mathbb{=E}\left[ W_{k\cdot}{\left( H\odot G \right)^{T}\mathcal{A}_{k\cdot}^{(3)}}^{T}-W_{k\cdot}\left( \sum_{i=1}^{I} \sum_{j=1}^{J} \mathcal{B}_{ijk}\left[ \left( {G_{i\cdot}}^{T}G_{i\cdot} \right)⊛\left( {H_{j\cdot}}^{T}H_{j\cdot} \right) \right] \right){W_{k\cdot}}^{T}-\frac{W_{k\cdot}diag\left( \boldsymbol{\lambda} \right){W_{k\cdot}}^{T}}{2} \right]+const\mathbb{=E}\left[ -\frac{W_{k\cdot}\left( 2\sum_{i=1}^{I} \sum_{j=1}^{J} \mathcal{B}_{ijk}\left[ \left( {G_{i\cdot}}^{T}G_{i\cdot} \right)⊛\left( {H_{j\cdot}}^{T}H_{j\cdot} \right) \right]+diag\left( \boldsymbol{\lambda} \right) \right){W_{k\cdot}}^{T}}{2}+W_{k\cdot}{\left( H\odot G \right)^{T}\mathcal{A}_{k\cdot}^{(3)}}^{T} \right]+const$ | (11) |
| --- | --- |

Therefore, the posterior approximation of $W_{k\cdot}$ also satisfies the multivariate Gaussian distribution, and its posterior parameters are obtained in the form of the above Gaussian distribution.

# S5 Text: The variational posterior distribution of $\boldsymbol{U}$

| $Lnq\left( U_{.r} \right)=\mathbb{E}_{q\left( \Theta\backslash U_{.r} \right)}\left[ Ln\left\{ P\left( G \vert U,S^{u},\sigma_{g} \right)P\left( U \vert\boldsymbol{\lambda} \right) \right\} \right]+const=\mathbb{E}\left[ \sum_{i=1}^{I} \sum_{r=1}^{R} \left( -\frac{\sigma_{g}\left[ \left( S_{i\cdot}^{u}U_{.r} \right)^{T}-{G_{ir}}^{T} \right]\left[ S_{i\cdot}^{u}U_{.r}-G_{ir} \right]}{2}-\frac{\lambda_{r}{U_{ir}}^{2}}{2} \right) \right]+const=\mathbb{E}\left[ \sum_{i_{n}=1}^{I_{n}} -\frac{\sigma_{g}{U_{.r}}^{T}{S_{i\cdot}^{u}}^{T}S_{i\cdot}^{u}U_{.r}-2\sigma_{g}{U_{.r}}^{T}{S_{i\cdot}^{u}}^{T}G_{ir}}{2}-\frac{\lambda_{r}{U_{ir}}^{2}}{2} \right]+const=\mathbb{E}\left[ -\frac{\sigma_{g}{U_{.r}}^{T}\left[ {S^{u}}^{T}S^{u} \right]U_{.r}-2\sigma_{g}{U_{.r}}^{T}{S^{u}}^{T}G_{\cdot r}}{2}-\frac{\lambda_{r}{U_{.r}}^{T}U_{.r}}{2} \right]+const=\mathbb{E}\left[ -\frac{{U_{.r}}^{T}\left[ \sigma_{g}{S^{u}}^{T}S^{u}+\lambda_{r}E_{I} \right]U_{.r}-2\sigma_{g}{U_{.r}}^{T}{S^{u}}^{T}G_{\cdot r}}{2} \right]+const$ | (12) |
| --- | --- |

Hence, we observe that $q\left( U_{.r} \right)$ is also a multivariate Gaussian distribution and the posterior parameters can be easily obtained from the above Gaussian form.

# S6 Text: The variational posterior distribution of $\boldsymbol{\lambda}$

| $Lnq\left( \boldsymbol{\lambda} \right)=\mathbb{E}_{q\left( \Theta\backslash\boldsymbol{\lambda} \right)}\left[ Ln\left\{ P\left( U \vert\boldsymbol{\lambda} \right)P\left( V \vert\boldsymbol{\lambda} \right)P\left( W \vert\boldsymbol{\lambda} \right)P\left( \boldsymbol{\lambda} \vert\alpha,\beta\right) \right\} \right]+const=\mathbb{E}\left[ \sum_{i=1}^{I} \left( \frac{Ln\left( \left\vert\Lambda\right\vert\right)}{2}-\frac{U_{i\cdot}\Lambda{U_{i\cdot}}^{T}}{2} \right)+\sum_{j=1}^{J} \left( \frac{Ln\left( \left\vert\Lambda\right\vert\right)}{2}-\frac{V_{j\cdot}\Lambda{V_{j\cdot}}^{T}}{2} \right)+\sum_{k=1}^{K} \left( \frac{Ln\left( \left\vert\Lambda\right\vert\right)}{2}-\frac{W_{k\cdot}\Lambda{W_{k\cdot}}^{T}}{2} \right)+\sum_{r=1}^{R} \left( \left( \alpha-1 \right)Ln\left( \lambda_{r} \right)-\beta\lambda_{r} \right) \right]+const=\mathbb{E}\left[ \frac{\left( I+J+K \right)}{2}\sum_{r=1}^{R} Ln\left( \lambda_{r} \right)-\frac{1}{2}\left( \sum_{r=1}^{R} \lambda_{r}\left( {U_{.r}}^{T}U_{.r}+{V_{.r}}^{T}V_{.r}+{W_{.r}}^{T}W_{.r} \right) \right)+\sum_{r=1}^{R} \left( \left( \alpha-1 \right)Ln\left( \lambda_{r} \right)-\beta\lambda_{r} \right) \right]+const=\mathbb{E}\left[ \sum_{r=1}^{R} \left[ \left( \frac{\left( I+J+K \right)}{2}+\alpha-1 \right)Ln\left( \lambda_{r} \right)-\left( \frac{\left( {U_{.r}}^{T}U_{.r}+{V_{.r}}^{T}V_{.r}+{W_{.r}}^{T}W_{.r} \right)}{2}+\beta\right)\lambda_{r} \right] \right]+const$ | (13) |
| --- | --- |

Therefore, $q\left( \lambda_{r} \right)$ follows the Gamma distribution.

# S7 Text: Prove theorem 1

| $\mathbb{E}\left[ \left\Vert G-S^{u}U \right\Vert^{2} \right]\mathbb{=E}\left[ tr\left[ \left( G-S^{u}U \right)\left( G^{T}-U^{T}\left( S^{u} \right)^{T} \right) \right] \right]\mathbb{=E}\left[ tr\left( GG^{T}+S^{u}UU^{T}\left( S^{u} \right)^{T}-2S^{u}UG^{T} \right) \right]=tr\left( \mathbb{E}\left[ GG^{T} \right]+S^{u}\mathbb{E}\left( UU^{T} \right)\left( S^{u} \right)^{T}-2S^{u}\mathbb{E}\left( U \right){\mathbb{E}\left( G \right)}^{T} \right)=tr\left( \mathbb{E}\left( G \right)\mathbb{E}\left( G \right)^{T}+diag\left[ \begin{matrix} tr\left( \Sigma\left( G_{1\cdot} \right) \right) & \cdots& tr\left( \Sigma\left( G_{I\cdot} \right) \right) \end{matrix} \right]+S^{u}\left\{ \mathbb{E}\left( U \right){\mathbb{E}\left( U \right)}^{T}+\sum_{r=1}^{R} \Sigma\left( U_{\cdot r} \right) \right\}\left( S^{u} \right)^{T} \right)=tr\left\{ \mathbb{E}\left( G \right)\mathbb{E}\left( G \right)^{T}+S^{u}\mathbb{E}\left( U \right){\mathbb{E}\left( U \right)}^{T}\left( S^{u} \right)^{T}-2S^{u}\mathbb{E}\left( U \right){\mathbb{E}\left( G \right)}^{T} \right\}+tr\left( diag\left[ \begin{matrix} tr\left( \Sigma\left( G_{1\cdot} \right) \right) & \cdots& tr\left( \Sigma\left( G_{I\cdot} \right) \right) \end{matrix} \right] \right)+tr\left( S^{u}\sum_{r=1}^{R} \Sigma\left( U_{\cdot r} \right)\left( S^{u} \right)^{T} \right)=\left\Vert\tilde{G}-S^{m}\tilde{U} \right\Vert^{2}+\sum_{i=1}^{I} tr\left( \Sigma\left( G_{i\cdot} \right) \right)+tr\left( S^{u}\sum_{r=1}^{R} \Sigma\left( U_{\cdot r} \right)\left( S^{u} \right)^{T} \right)$ | (14) |
| --- | --- |

Where, $\left\| \cdot\right\|$ represents the Frobenius norm of the matrix, $\tilde{G}$ and $\tilde{U}$ represent the posterior expectations of $G$ and $U$ respectively, and $tr\left( \cdot\right)$ represents the trace of the square matrix.

# S8 Text: Solution of local variational parameter $\boldsymbol{\xi}_{\boldsymbol{ijk}}$

| $L\left( \xi_{ijk} \right)=E\left[ Ln\left( h\left（ \xi_{ijk},G,H,W \right） \right) \right]\mathbb{=E}\left[ (c\mathcal{Y}_{ijk}+1-\mathcal{Y}_{ijk})\left\{ Ln\left[ \sigma\left( \xi_{ijk} \right) \right]-\frac{{\tilde{\mathcal{Y}}}_{ijk}+\xi_{ijk}}{2}-\lambda\left( \xi_{ijk} \right)\left( {{\tilde{\mathcal{Y}}}_{ijk}}^{2}-{\xi_{ijk}}^{2} \right) \right\} \right]+const$ | (15) |
| --- | --- |

Let $\mathbb{E}\left( {{\tilde{\mathcal{Y}}}_{ijk}}^{2} \right)=a^{2}$, $\xi=\xi_{ijk}$, and remove the constant term $\left( c\mathcal{Y}_{ijk}+1-\mathcal{Y}_{ijk} \right)$. Then, (15) becomes

| $\tilde{L}\left( \xi\right)=Ln\left[ \sigma\left( \xi\right) \right]-\frac{\xi}{2}-\lambda\left( \xi\right)\left( a^{2}-\xi^{2} \right)$ | (16) |
| --- | --- |

Since $c\mathcal{Y}_{ijk}+1-\mathcal{Y}_{ijk}>0$ is always true, $L\left( \xi\right)$ and $\tilde{L}\left( \xi\right)$ take the maximum value at the same position. Derivative of $L\left( \xi\right)$ with respect to $\xi$

| $\frac{d\tilde{L}\left( \xi\right)}{d\xi}=\frac{\sigma^{'}\left( \xi\right)}{\sigma\left( \xi\right)}-\frac{1}{2}-a^{2}\lambda^{'}\left( \xi\right)+\lambda^{'}\left( \xi\right)\xi^{2}+2\xi\lambda\left( \xi\right)$ | (17) |
| --- | --- |

Substituting the equations $\sigma^{'}\left( \xi\right)=\sigma\left( \xi\right)\left( 1-\sigma\left( \xi\right) \right)$, $2\xi\lambda\left( \xi\right)=\sigma\left( \xi\right)-\frac{1}{2}$ into (17), we get:

$$1-\sigma\left( \xi\right)-\frac{1}{2}-a^{2}\lambda^{'}\left( \xi\right)+\lambda^{'}\left( \xi\right)\xi^{2}+\sigma\left( \xi\right)-\frac{1}{2}=0$$

The above formula is further simplified to

$$\lambda^{'}\left( \xi\right)\left( \xi^{2}-a^{2} \right)=0$$

Therefore, the value of the local variation parameter $\xi_{ijk}$ is

| ${\xi_{ijk}}^{2}\mathbb{=E}\left( {{\bar{\mathcal{Y}}}_{ijk}}^{2} \right)\mathbb{=E}\left( \left\langle G_{i\cdot},H_{j\cdot},W_{k\cdot} \right\rangle^{2} \right)\mathbb{=E}\left[ \left( \sum_{r=1}^{R} G_{ir}H_{jr}W_{kr} \right)^{2} \right]\mathbb{=E}\left[ \left\{ \sum_{r} \left( G_{i\cdot}⊛H_{j\cdot}⊛W_{k\cdot} \right)_{r} \right\}^{2} \right]\mathbb{=E}\left[ \sum_{r_{1},r_{2}} \left( \left( G_{i\cdot}⊛H_{j\cdot}⊛W_{k\cdot} \right) \right)_{r_{1}}\left( \left( G_{i\cdot}⊛H_{j\cdot}⊛W_{k\cdot} \right) \right)_{r_{2}} \right]\mathbb{=E}\left[ \sum_{r_{1},r_{2}} \left\{ \left( \left( G_{i\cdot}⊛H_{j\cdot}⊛W_{k\cdot} \right) \right)^{T}\left( \left( G_{i\cdot}⊛H_{j\cdot}⊛W_{k\cdot} \right) \right) \right\}_{r_{1},r_{2}} \right]\mathbb{=E}\left[ \sum_{r_{1},r_{2}} \left\{ \left( {G_{i\cdot}}^{T}G_{i\cdot} \right)⊛\left( {H_{j\cdot}}^{T}H_{j\cdot} \right)⊛\left( {W_{k\cdot}}^{T}W_{k\cdot} \right) \right\}_{r_{1},r_{2}} \right]\mathbb{=E}\left[ \left\langle{G_{i\cdot}}^{T}G_{i\cdot},{H_{j\cdot}}^{T}H_{j\cdot},{W_{k\cdot}}^{T}W_{k\cdot} \right\rangle\right]=\left\langle\mathbb{E}\left[ {G_{i\cdot}}^{T}G_{i\cdot} \right]\mathbb{,E}\left[ {H_{j\cdot}}^{T}H_{j\cdot} \right]\mathbb{,E}\left[ {W_{k\cdot}}^{T}W_{k\cdot} \right] \right\rangle$ | (18) |
| --- | --- |

where $\left\langle\cdot\right\rangle$ is the generalized inner product symbol, which represents the summation of the corresponding element products [1].

# S9 Text: Result statistics of $\boldsymbol{CV}_{\boldsymbol{triplet}}$ scenarios

Under ${CV}_{triplet}$, for each fold of five-fold cross-validation, we set 20 random seeds to select the test set. The mean and variance of the prediction indicators of all methods are shown in S4 Table.

From Table S4, KBLTDARD achieves the best mean value in all indicators of HMDD v2.0 and HMDD v3.2, and has a small variance, indicating that KBLTDARD has a certain degree of robustness. The box plot of the prediction results of KBLTDARD under 20 random seeds is shown in S1 Fig.

In addition, we performed a paired Wilcoxon rank sum test between KBLTDARD and other prediction models in terms of AUC, AUPR, and F1 score, and the results are shown in S5 Table. Apparently, KBLTDARD significantly outperforms other prediction models at 95% confidence level (p-value < 0.05) on all datasets. This once again demonstrates the superiority of KBLTDARD in miRNA-disease-type triad prediction.

References

[1] Q. Zhao, L. Zhang, and A. Cichocki, “Bayesian CP Factorization of Incomplete Tensors with Automatic Rank Determination,” *IEEE Trans Pattern Anal Mach Intell,* vol. 37, no. 9, pp. 1751-63, Sep, 2015.
