# Supplementary figures and images for: Kernel Bayesian logistic tensor decomposition with automatic rank determination for predicting multiple types of miRNA-disease associations
Source: PLoS Comput Biol. 2024 Jul 8;20(7):e1012287. doi: 10.1371/journal.pcbi.1012287 (PMC11257412; doi:10.1371/journal.pcbi.1012287)

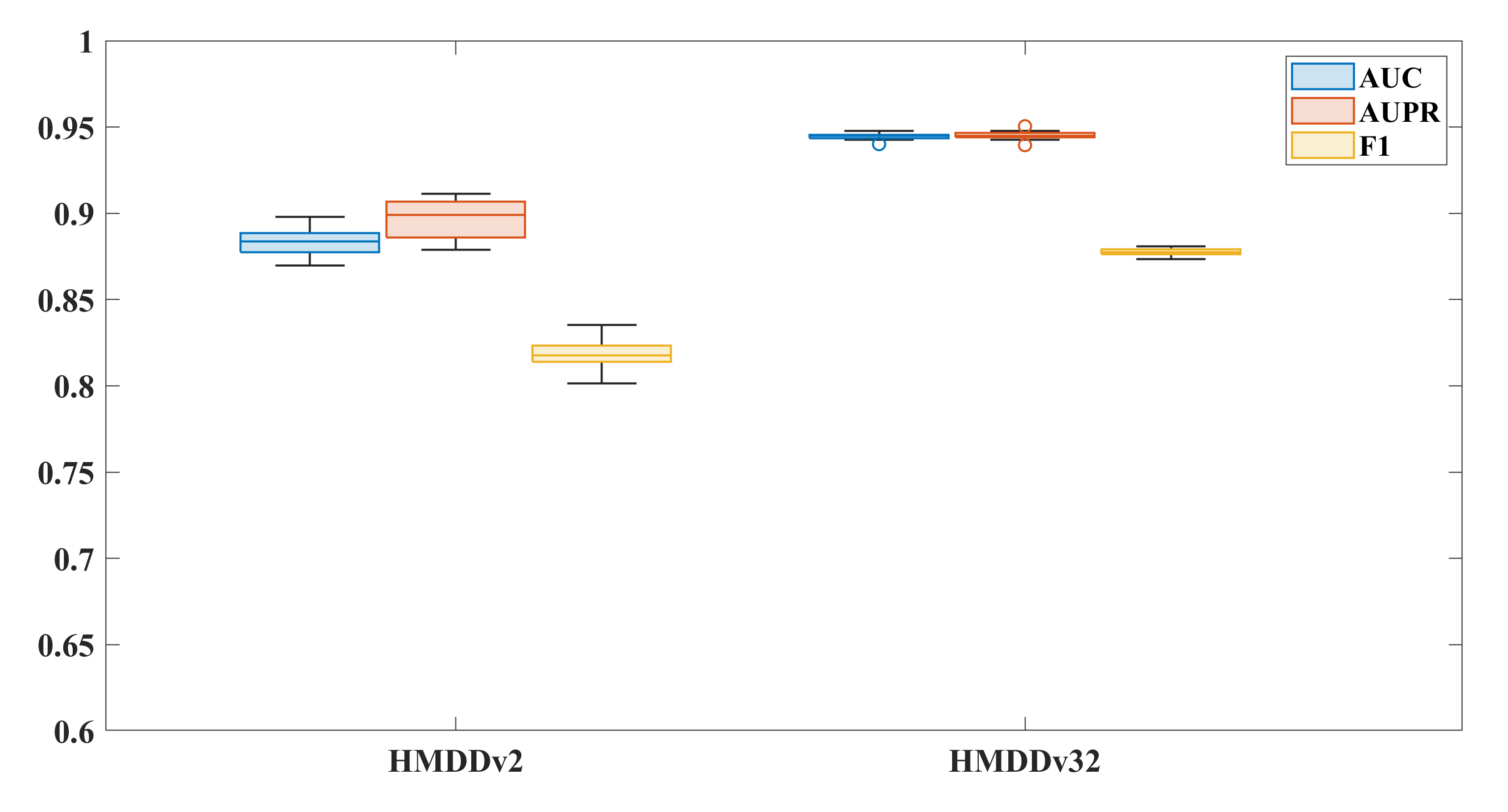

Supplement: S1 Fig — (TIF) [file pcbi.1012287.s002.tif]
