# Supplementary material for: Kernel Bayesian logistic tensor decomposition with automatic rank determination for predicting multiple types of miRNA-disease associations
Source: PLoS Comput Biol. 2024 Jul 8;20(7):e1012287. doi: 10.1371/journal.pcbi.1012287 (PMC11257412; doi:10.1371/journal.pcbi.1012287)
Supplement: S1 Table — (DOCX) [file pcbi.1012287.s003.docx]

**S1 Table**. Myocardial Infarction-related miRNAs and association types predicted by KBLTDARD.

| Rank | MiRNA | Type | PMID | Rank | MiRNA | Type | PMID |
| --- | --- | --- | --- | --- | --- | --- | --- |
| 1 | hsa-mir-21 | circulation | 26337652 | 11 | hsa-mir-206 | circulation | 19245789 |
| 2 | hsa-mir-155 | tissue | 19581315 | 12 | hsa-mir-150 | circulation | 23967079 |
| 3 | hsa-mir-21 | tissue | 19706597 | 13 | hsa-mir-1 | circulation | 23630629 |
| 4 | hsa-mir-155 | circulation | 28618412 | 14 | hsa-mir-122 | tissue | Unconfirmed |
| 5 | hsa-mir-21 | target | 19336275 | 15 | hsa-mir-150 | tissue | 20075508 |
| 6 | hsa-mir-146a | tissue | Unconfirmed | 16 | hsa-mir-1 | tissue | 24046434 |
| 7 | hsa-mir-146a | circulation | 26337652 | 17 | hsa-mir-210 | circulation | 27346801 |
| 8 | hsa-mir-146a | target | 30362610 | 18 | hsa-mir-133a | tissue | Unconfirmed |
| 9 | hsa-mir-155 | target | 26258537 | 19 | hsa-mir-223 | circulation | 22813605 |
| 10 | hsa-mir-133a | circulation | 29324314 | 20 | hsa-mir-122 | circulation | Unconfirmed |
