# Supplementary material for: Kernel Bayesian logistic tensor decomposition with automatic rank determination for predicting multiple types of miRNA-disease associations
Source: PLoS Comput Biol. 2024 Jul 8;20(7):e1012287. doi: 10.1371/journal.pcbi.1012287 (PMC11257412; doi:10.1371/journal.pcbi.1012287)
Supplement: S2 Table — (DOCX) [file pcbi.1012287.s004.docx]

**S2 Table**. Prostate Neoplasms-related miRNAs and association types predicted by KBLTDARD.

| Rank | MiRNA | Type | PMID | Rank | MiRNA | Type | PMID |
| --- | --- | --- | --- | --- | --- | --- | --- |
| 1 | hsa-mir-21 | tissue | 16461460 | 11 | hsa-mir-203 | tissue | 22391564 |
| 2 | hsa-mir-206 | tissue | Unconfirmed | 12 | hsa-mir-34a | tissue | 22719071 |
| 3 | hsa-mir-34c | tissue | 20162671 | 13 | hsa-mir-373 | circulation | Unconfirmed |
| 4 | hsa-mir-34b | tissue | 28039468 | 14 | hsa-mir-29c | tissue | Unconfirmed |
| 5 | hsa-mir-146a | tissue | 16461460 | 15 | hsa-mir-29a | tissue | 21592394 |
| 6 | hsa-mir-183 | tissue | 20873592 | 16 | hsa-mir-21 | target | 19302977 |
| 7 | hsa-mir-182 | tissue | 20873592 | 17 | hsa-mir-21 | genetics | Unconfirmed |
| 8 | hsa-mir-210 | tissue | 30109809 | 18 | hsa-mir-221 | tissue | 18949015 |
| 9 | hsa-mir-200b | tissue | 20539944 | 19 | hsa-mir-429 | tissue | 20539944 |
| 10 | hsa-mir-21 | circulation | 21274675 | 20 | hsa-mir-34b | target | 26107383 |
