# Supplementary material for: Kernel Bayesian logistic tensor decomposition with automatic rank determination for predicting multiple types of miRNA-disease associations
Source: PLoS Comput Biol. 2024 Jul 8;20(7):e1012287. doi: 10.1371/journal.pcbi.1012287 (PMC11257412; doi:10.1371/journal.pcbi.1012287)
Supplement: S3 Table — (DOCX) [file pcbi.1012287.s005.docx]

**S3 Table**. Pancreatic Neoplasms-related miRNAs and association types predicted by KBLTDARD.

| Rank | MiRNA | Type | PMID | Rank | MiRNA | Type | PMID |
| --- | --- | --- | --- | --- | --- | --- | --- |
| 1 | hsa-mir-21 | tissue | 16461460 | 11 | hsa-mir-221 | tissue | 16461460 |
| 2 | hsa-mir-21 | circulation | 21139804 | 12 | hsa-mir-183 | tissue | 22042419 |
| 3 | hsa-mir-155 | tissue | 16966691 | 13 | hsa-mir-375 | tissue | Unconfirmed |
| 4 | hsa-mir-146a | tissue | 16461460 | 14 | hsa-mir-9 | tissue | 29169171 |
| 5 | hsa-mir-21 | target | 21376256 | 15 | hsa-mir-200b | tissue | 19030927 |
| 6 | hsa-mir-122 | tissue | 22850622 | 16 | hsa-mir-29b | tissue | 22820191 |
| 7 | hsa-mir-182 | tissue | 22042419 | 17 | hsa-mir-200a | tissue | 22114139 |
| 8 | hsa-mir-155 | circulation | 19106647 | 18 | hsa-mir-15a | tissue | 19551852 |
| 9 | hsa-mir-210 | tissue | 19551852 | 19 | hsa-mir-125a | tissue | Unconfirmed |
| 10 | hsa-mir-223 | tissue | 16461460 | 20 | hsa-mir-29a | tissue | Unconfirmed |
