# Supplementary material for: Kernel Bayesian logistic tensor decomposition with automatic rank determination for predicting multiple types of miRNA-disease associations
Source: PLoS Comput Biol. 2024 Jul 8;20(7):e1012287. doi: 10.1371/journal.pcbi.1012287 (PMC11257412; doi:10.1371/journal.pcbi.1012287)
Supplement: S4 Table — (DOCX) [file pcbi.1012287.s006.docx]

S4 Table. Statistics of predictions for all models under ${CV}_{triplet}$.

| Method | HMDD v2.0 | | |  | HMDD v3.2 | | |
| --- | --- | --- | --- | --- | --- | --- | --- |
|  | AUPR | AUC | F1 |  | AUPR | AUC | F1 |
| TFAI | 0.8539  ±0.0087 | 0.8194  ±0.0063 | 0.7742  ±0.0061 |  | 0.9211  ±0.0028 | 0.9046  ±0.0021 | 0.8498  ±0.0025 |
| FBCPARD | 0.7710  ±0.0073 | 0.6764  ±0.0049 | 0.6746  ±0.0038 |  | 0.8741  ±0.0028 | 0.8239  ±0.0016 | 0.8027  ±0.0019 |
| TDRC | 0.8602  ±0.0087 | 0.8264  ±0.0069 | 0.7754  ±0.0054 |  | 0.9243  ±0.0029 | 0.9121  ±0.0022 | 0.8517  ±0.0029 |
| WeightTDAIGN | 0.8170  ±0.0065 | 0.7434  ±0.0043 | 0.724  ±0.0059 |  | 0.9099  ±0.0029 | 0.8841  ±0.002 | 0.8405  ±0.0024 |
| TFLP | 0.8220  ±0.0063 | 0.7639  ±0.0115 | 0.7771  ±0.0078 |  | 0.8193  ±0.0021 | 0.7447  ±0.0044 | 0.7774  ±0.0028 |
| SPLDHyperAWNTF | 0.8817  ±0.0086 | 0.8646  ±0.0061 | 0.8025  ±0.0064 |  | 0.9294  ±0.0028 | 0.9214  ±0.002 | 0.8569  ±0.0021 |
| KBLTD_ARD | 0.8966  ±0.0106 | 0.8835  ±0.0079 | 0.8185  ±0.0078 |  | 0.9452  ±0.0023 | 0.9445  ±0.0016 | 0.8775  ±0.002 |
