# Supplementary material for: Kernel Bayesian logistic tensor decomposition with automatic rank determination for predicting multiple types of miRNA-disease associations
Source: PLoS Comput Biol. 2024 Jul 8;20(7):e1012287. doi: 10.1371/journal.pcbi.1012287 (PMC11257412; doi:10.1371/journal.pcbi.1012287)
Supplement: S5 Table — (DOCX) [file pcbi.1012287.s007.docx]

S5 Table. The P-value of the Wilcoxon rank sum test for pairing KBLTDARD with other models.

|  | TFAI | FBCPARD | TDRC | WeightTDAIGN | TFLP | SPLDHyperAWNTF |
| --- | --- | --- | --- | --- | --- | --- |
| HMDDv2 | 5.2393×10^-10^ | 3.5566×10^-21^ | 1.6030×10^-8^ | 1.2588×10^-17^ | 6.7460×10^-16^ | 1.0165×10^-4^ |
| HMDDv32 | 1.5543×10^-7^ | 6.8495×10^-20^ | 1.5543×10^-7^ | 1.5106×10^-7^ | 3.5566×10^-21^ | 1.5543×10^-7^ |
